# A lightweight data-driven spiking neuronal network model of *Drosophila* olfactory nervous system with dedicated hardware support

**DOI:** 10.3389/fnins.2024.1384336

**Published:** 2024-06-26

**Authors:** Takuya Nanami, Daichi Yamada, Makoto Someya, Toshihide Hige, Hokto Kazama, Takashi Kohno

**Affiliations:** ^1^Institute of Industrial Science, The University of Tokyo, Meguro Ku, Tokyo, Japan; ^2^Department of Biology, University of North Carolina at Chapel Hill, Chapel Hill, NC, United States; ^3^RIKEN Center for Brain Science, Wako, Saitama, Japan; ^4^Department of Cell Biology and Physiology, University of North Carolina at Chapel Hill, Chapel Hill, NC, United States; ^5^Integrative Program for Biological and Genome Sciences, University of North Carolina at Chapel Hill, Chapel Hill, NC, United States

**Keywords:** spiking neuronal network, PQN model, *Drosophila*, field-programmable gate array, olfactory nervous system

## Abstract

Data-driven spiking neuronal network (SNN) models enable *in-silico* analysis of the nervous system at the cellular and synaptic level. Therefore, they are a key tool for elucidating the information processing principles of the brain. While extensive research has focused on developing data-driven SNN models for mammalian brains, their complexity poses challenges in achieving precision. Network topology often relies on statistical inference, and the functions of specific brain regions and supporting neuronal activities remain unclear. Additionally, these models demand huge computing facilities and their simulation speed is considerably slower than real-time. Here, we propose a lightweight data-driven SNN model that strikes a balance between simplicity and reproducibility. The model is built using a qualitative modeling approach that can reproduce key dynamics of neuronal activity. We target the *Drosophila* olfactory nervous system, extracting its network topology from connectome data. The model was successfully implemented on a small entry-level field-programmable gate array and simulated the activity of a network in real-time. In addition, the model reproduced olfactory associative learning, the primary function of the olfactory system, and characteristic spiking activities of different neuron types. In sum, this paper propose a method for building data-driven SNN models from biological data. Our approach reproduces the function and neuronal activities of the nervous system and is lightweight, acceleratable with dedicated hardware, making it scalable to large-scale networks. Therefore, our approach is expected to play an important role in elucidating the brain's information processing at the cellular and synaptic level through an analysis-by-construction approach. In addition, it may be applicable to edge artificial intelligence systems in the future.

## 1 Introduction

Elucidating the mechanisms underlying information processing in the brain represents a great challenge in neuroscience. In parallel to collecting data with experiments, building brain models has proven to be a powerful approach to enable *in-silico* analysis and provide a framework for understanding information processing in the brain. Macroscopic models (Kawato, 1999; Frank et al., 2001; Norman and O'Reilly, 2003; Walther and Koch, 2006) describe information flow at the functional level and present an overview of neural processing. In contrast, spiking neuronal network (SNN) models emulate the brain at the cellular and synaptic level and provide their *in-silico* counterparts, which are more tractable and easier to manipulate. From an engineering perspective, properly built SNN models are expected to be capable of intelligent information processing equivalent to the brain. Silicon neuronal network (SiNN) chips, which are highly power-efficient neuromorphic hardware optimized for SNN models, have already been developed (Merolla et al., 2014; Qiao et al., 2015; Davies et al., 2018). Therefore, they have great potential for next-generation artificial intelligence (AI) applications.

The structure of the brain is highly diverse, which makes it demanding to capture the comprehensible rules about the network topology. In addition, a wide variety of neuronal and synaptic properties has been reported. The data-driven approach intends to replicate the brain by semi-automatically incorporating vast amounts of anatomical and physiological data. Several large-scale data-driven SNN models (Markram et al., 2015; Bezaire et al., 2016; Ecker et al., 2020) that reproduce a part of the mammalian cortex and hippocampus have been built. They were designed to replicate the network topology, neuronal anatomy and electrophysiology, and synaptic properties, and they successfully reproduced the characteristic spiking activities seen in the target regions. However, in mammalian brains, the considerable number of neurons makes it challenging to measure the exact connection topology between the neurons. Hence, the network topology was inferred based on statistical data. In addition, because each brain region closely interacts with various other brain regions, it is not trivial to understand the specific function of the target region. Generally, data-driven models employ the ionic-conductance-based neuronal models, which can reproduce arbitrary electrophysiological properties but incur enormous computational costs. For example, the model in Bezaire et al. (2016) runs on a supercomputer consisting of 3,488 processors, and its simulation speed is 1,600 times slower than real-time. Moreover, these models are not suitable for implementation on SiNNs because they involve complex calculation processes that require enormous circuit resources.

In this study, we built a data-driven SNN model for the olfactory nervous system of *Drosophila melanogaster* (fruit fly). The system is a relatively small (~2,200 neurons) network having a known function, whose complete network topology, or connectome, is available. The electrophysiological activity of neurons was reproduced by using the piecewise quadratic neuron (PQN) model, which is a lightweight neuron model suitable for digital arithmetic circuit implementations (Nanami and Kohno, 2016a,b, 2023; Nanami et al., 2016, 2017, 2018).

The PQN model was adopted to reduce the computational cost and enable the SNN model to be run on a SiNN chip. It focuses on reproducing the key dynamics behind neuronal activities with lightweight calculations. The model is designed using the dimension reduction techniques of nonlinear dynamics such that the dynamical structure of the activity of the target neuron is preserved. Unlike integrate-and-fire (I&F) based models, such as the leaky I&F model, Izhikevich (IZH) model (Izhikevich, 2003), and adaptive exponential I&F model (Brette and Gerstner, 2005), the dynamics in the neuronal spike are not replaced by a resetting of the membrane potential. I&F-based models are generally more lightweight than the PQN model. However, they have been reported to have limitations in the reproducibility of neuronal activities. For example, because their spike amplitudes are fixed, they cannot reproduce the propagation of spike intensity observed in some brain regions including the hippocampus (Alle and Geiger, 2006). In addition, the IZH model can only reproduce spiking within a limited range of stimulus intensities (Nanami and Kohno, 2016b). Furthermore, the phase-resetting curve of the Class *II* mode in Hodgkin's classification (Hodgkin, 1948) of the IZH and AdEx models differs from the typical shape (Nanami and Kohno, 2023). In addition to the aforementioned advantages, the PQN model supports the efficient implementation on digital arithmetic circuits. Thus, the SNN model can be executed efficiently (power and speed) with a SiNN on field-programmable gate arrays (FPGAs) and application-specific integrated circuits (ASICs). The results in this study were obtained using a SiNN on an entry-level low-cost FPGA chip to demonstrate its potential for low-power brain-morphic artificial intelligence (AI) applications.

In recent years, brain-inspired AI has become popular, where spike-based machine learning (Yang and Chen, 2023a,b; Yang et al., 2023a,b) is studied mainly using I&F-based models. These studies built massively parallel information processing systems inspired by the brain's structure to enable advanced and robust information processing with low power consumption. In contrast, here we aim to provide an *in silico* platform that more faithfully reproduces neuronal connectivity and information processing in brain microcircuits, which is distinct from the objective of brain-inspired AI.

The fruit fly brain comprises 100,000 neurons. Moreover, its connectome was recently revealed (Scheffer et al., 2020). It is compact compared to the mammalian brain but capable of complex information processing. Its olfactory nervous system consists of brain regions including the antennal lobe and the mushroom body, the anatomy and physiology of which have been widely studied (Wilson, 2013; Modi et al., 2020). The function and activity of each type of neuron in these regions are better characterized in the context of sensory input and behavioral output than those of the mammalian cortex and hippocampus, enabling us to adequately verify the reproducibility of the model. However, previous modeling studies (Wessnitzer et al., 2012; Faghihi et al., 2017; Kennedy, 2019) (not data-driven) of the olfactory nervous system used simplified I&F-based neuron models, which did not fully reproduce the electrophysiological properties of each type of neurons. Specifically, they did not reproduce the characteristic spiking activities seen in the olfactory nervous system including (1) odor-evoked oscillatory firing in the projection neurons (PNs) and local neurons (LNs) (Tanaka et al., 2009), (2) absence of oscillations in Kenyon cells (KCs) (Turner et al., 2008), (3) different contributions of LN subclasses to the formation of oscillations (Tanaka et al., 2009), and (4) temporal dynamics of firing in mushroom body output neurons (MBONs) (Hige et al., 2015). Thus, it is uncertain whether they accurately capture information processing mechanisms in the olfactory nervous system. More sophisticated, ionic-conductance-based SNN models of the insect brain (Bazhenov et al., 2001a,b) had been built for the antennal lobe of locust. However, they were not data-driven and did not reproduce most of the aforementioned characteristics of spiking activities. This is likely because they modeled only PNs and LNs, and also lacked electrophysiological data on identified neurons. Here we built a model of a fly olfactory system incorporating the connectome data as well as neuronal and synaptic electrophysiological properties of neurons. Our model successfully reproduced not only the aforementioned characteristic spiking activities (1)–(4) of the constituent cells, but also olfactory associative plasticity, the primary function of the olfactory system. Although we did not intend to implement every single known neuron or connection in our model, this study lays a foundation for building lightweight data-driven SNN models and is expected to aid in understanding the brain and developing brain-morphic AI systems.

## 2 Methods

### 2.1 Network model

**Figure 1:**
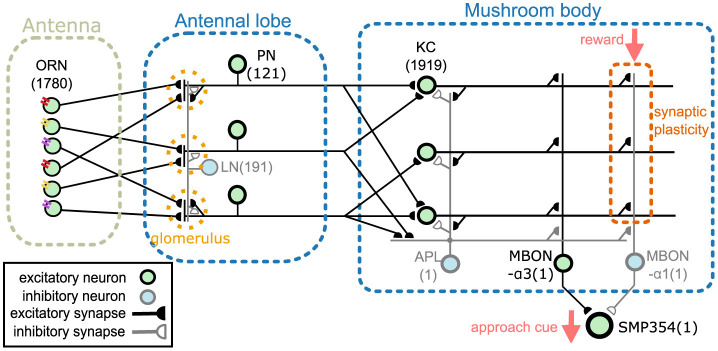
Network overview. The network comprises an antenna, the antennal lobe, and the mushroom body. ORNs, PNs, KCs, and MBON-α3 are excitatory neurons, whereas LNs, APL, and MBON-α1 are inhibitory neurons. SMP354 neuron receives excitatory and inhibitory input from the two MBONs and produces the approach cue. KC > MBON-α1 synapses can express synaptic plasticity, which is driven by the reward signal.

This section provides an overview of the proposed network model. The model comprises an antenna, the antennal lobe, and the mushroom body (Figure 1). The antenna contains olfactory receptor neurons (ORNs), and the antennal lobe contains LNs and PNs (Stocker et al., 1990). KCs, an anterior paired lateral neuron (APL), and MBONs are present in the mushroom body (Aso et al., 2014a). The two MBONs, MBON-α1 and MBON-α3, project to SMP354 neuron, whose excitatory activity can trigger a series of olfactory approach behaviors including upwind steering and locomotion (Aso et al., 2023). ORNs, PNs, KCs, and MBON-α3 are cholinergic (Yasuyama and Salvaterra, 1999; Kazama and Wilson, 2008; Tanaka et al., 2008; Barnstedt et al., 2016) and form excitatory synapses. Some LNs are cholinergic (Shang et al., 2007) or glutamatergic (Das et al., 2011) and are considered as sources of excitatory or inhibitory input (Olsen et al., 2007; Shang et al., 2007). However, most LNs are GABAergic (Python and Stocker, 2002; Wilson and Laurent, 2005) and have been shown to provide inhibitory input (Olsen and Wilson, 2008; Root et al., 2008). Thus, in this model, all LNs were set as inhibitory. APL is GABAergic (Tanaka et al., 2008) and inhibitory, whereas MBON-α1 is glutaminergic (Aso et al., 2014a) and inhibitory. The numbers in Figure 1 represent the numbers of neurons implemented in the model. Synaptic connections were determined using the connectome database hemibrain v1.2.1 (HEM, 2020; Scheffer et al., 2020). Since this model targets the olfactory nervous system of the right hemisphere, we first obtained the number of neurons of each type in the right hemisphere from the hemibrain server using the NC function of the neuprint-python library. We then determined the connections between neurons using the fetch_neurons function of the neuprint-python that returns the number of synaptic connections between neurons. Connections with more than ten synapses were assumed to have sufficient strength, and their weight *w* was set to 1. Otherwise, *w* was set to 0. Note that the connections of LNs were determined based on a previous study (Seki et al., 2010).

A variety of LN subclasses were reported (Chou et al., 2010; Seki et al., 2010) identified four subclasses, each with different spiking properties. However, the connectome database (HEM, 2020; Scheffer et al., 2020) does not describe which subclass each LN belongs to. Therefore, the connection of LNs was determined based on Seki et al. (2010) where the probabilities that each LN subclass has a connection to a certain glomerulus were shown. In the antennal lobe, glomeruli are neuropils comprising axons and dendrites of PNs, LNs, and ORNs. ORNs and PNs are generally connected to only one glomerulus. We first determined the subclasses to which 191 LNs belong. As the proportion of each subclass is unknown, we set the number of NP2426_class1 to 47 and the remaining to 48 to ensure that the distribution of subclasses was as even as possible. Next, for each LN, we randomly determined whether each LN innervates each glomerulus according to the innervation probabilities shown in Seki et al. (2010). If an ORN/PN and LN innervate the same glomerulus, the ORN/PN was assumed to make a synaptic connection onto the LN, and the synaptic weight *w* was set to 1. For example, LNs NP1227_class1 connect to glomerulus DA1 with a probability of 75% (Seki et al., 2010). Based on this probability, we determined whether each LN NP1227_class1 connects to glomerulus DA1.

Each ORN expresses one of the olfactory receptors, each of which has different odor selectivity. A previous study (D.Münch and Galizia, 2016) described a correspondence table between glomerulus and olfactory receptor (OR) types. We used this table and the glomerulus type for each ORN listed in the connectome database to determine the OR type for each ORN. When multiple OR types were assigned to a single glomerulus type, one OR was randomly selected.

**Figure 2:**
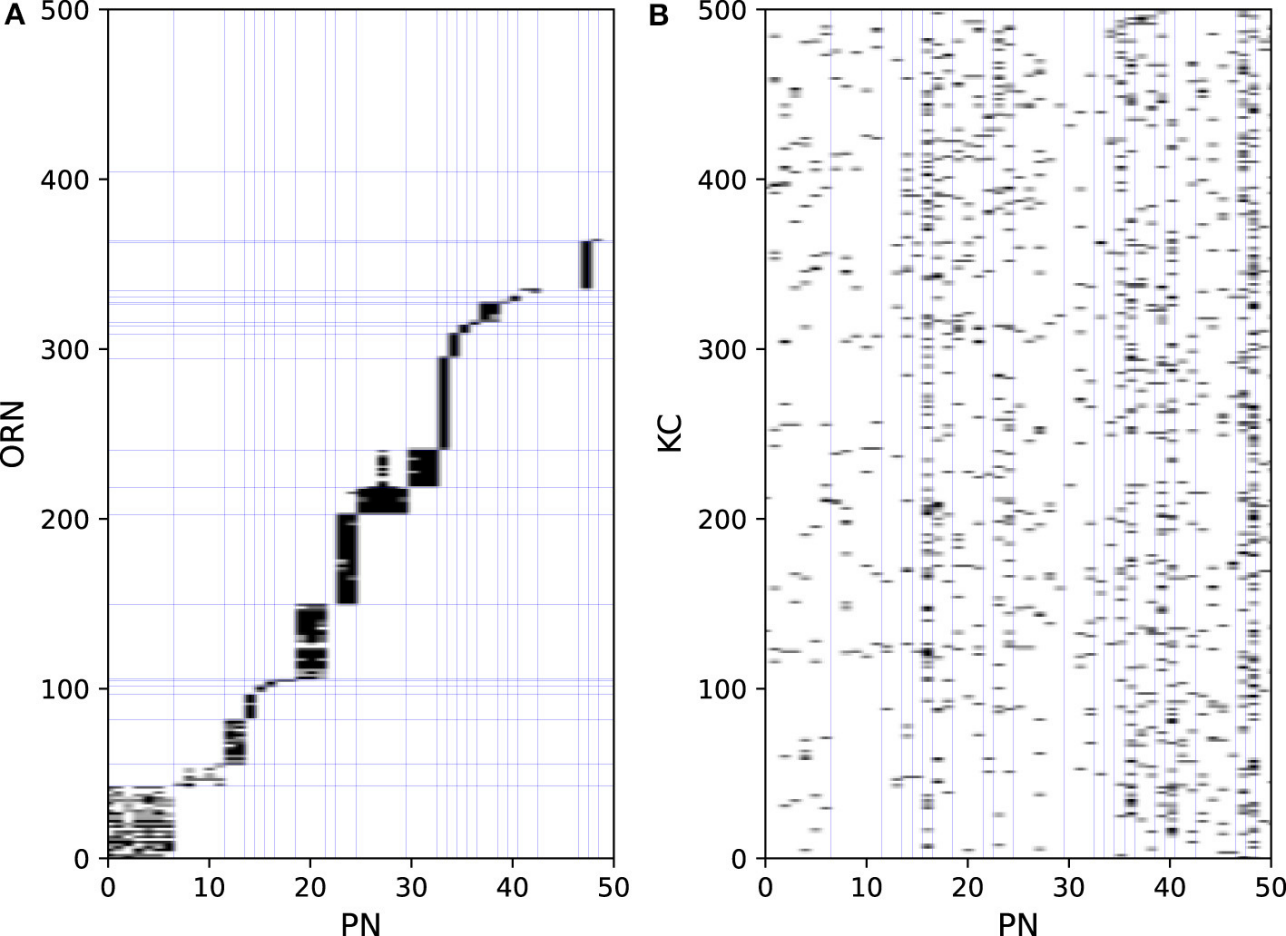
Synaptic connections from ORNs to PNs **(A)** and PNs to KCs **(B)**.

Odors are first detected by ORNs on the antenna. ORNs express one of the ORs, each possessing different odor selectivity. ORNs extend their axons to the antennal lobe and project to PNs and LNs. As the firing activities of ORNs are the input data, they are not modeled. Figure 2A shows part of the connection structure from ORNs to PNs in the model. The black squares represent the presence of connections. On average, each ORN projects to 1.6 PNs, and each PN receives input from 24.0 ORNs. ORNs and PNs were sorted based on their glomerulus type, the borders of which are represented by blue lines. ORNs generally project to all the PNs in the same glomerulus type (Kazama and Wilson, 2009). This convergent projection is considered (Bhandawat et al., 2007) to enable PNs to produce reliable output by averaging the input from a large number of ORNs, whereas the responses of ORNs to odors are noisy and unreliable (Stocker et al., 1990). LNs receive inputs from a wide range of ORNs and extensively inhibit PNs and LNs. On average, each LN receives input from 1337.4 ORNs and inhibits 90.9 PNs. LNs are considered to contribute to the gain control of the input from ORNs (Olsen and Wilson, 2008) and to the generation of oscillations in the antennal lobe (Tanaka et al., 2009).

PNs extend their axons to the entrance of the mushroom body, where they provide excitatory input to KCs. On average, each PN projects to 67.6 KCs, and each KC receives input from 4.2 PNs. Figure 2B shows part of the connection structure from PNs to KCs in the model. In contrast to the connections between ORNs and PNs, there is no regularity in the connections between PNs and KCs, which was confirmed in a previous study (Caron et al., 2013). APL receives input from almost all KCs and PNs and returns inhibitory feedback to KCs and MBONs.

There are 28 MBONs, or 44 including atypical MBONs (Li et al., 2020), at least some of which signal either positive or negative valence (Aso et al., 2014b). While how MBON signals are further processed by the downstream circuits to determine the behavioral output is still largely elusive, the connectome study discovered that postsynaptic neurons of the MBONs typically receive synaptic input from more than one type of MBONs (Li et al., 2020), suggesting that valence signals could be integrated by those neurons. A recent study characterized such a circuit motif experimentally (Aso et al., 2023). A cluster of 8–10 neurons named UpWind Neurons (UpWiNs) directly and indirectly integrates excitatory and inhibitory input from MBON-α3 and MBON-α1, respectively. Direct optogenetic activation of MBON-α3 induces upwind locomotion, which can be interpreted as an olfactory approach behavior (Matheson et al., 2021), while activation of MBON-α1 does not induce such behavior (Aso et al., 2023). Experiments using compartment-specific optogenetic activation of dopaminergic neurons demonstrated that α3 and α1 are an aversive- and appetitive-memory compartment, respectively (Aso and Rubin, 2016). Moreover, optogenetic activation of UpWiNs triggers robust upwind locomotion (Aso et al., 2023). Thus, the UpWiN cluster is one of the sites where signals of opposite memory valence are integrated and translated into olfactory navigation behavior. Since neurons in the UpWiN cluster are heterogeneous in their anatomy and connectivity, in our model, we focused on one of the neurons, SMP354, (bodyId in hemibrain is 390003153), which receives direct synaptic input from both MBON-α3 and MBON-α1. Because there is no specific genetic driver to label this particular neuron, we were unable to use experimentally determined electrophysiological parameters for this neuron. Although our model is simplified in terms of the readout mechanism of the mushroom body signals, we believe that the SMP354 circuit represents one of the common motifs that interpret the population signals of MBONs.

If a reward or punishment is given to a fly with an odor, the fly will learn to approach or avoid that odor thereafter (Tully and Quinn, 1985). Multiple studies (Cohn et al., 2015; Hige et al., 2015; Owald et al., 2015) indicate that this olfactory associative learning is caused by long-term depression of KC>MBON synapses. In the case of the circuit associated with UpWiNs, it has been experimentally demonstrated that induction of plasticity in α1, which mimics appetitive conditioning, depresses olfactory responses of MBON-α1. This in turn potentiates responses of UpWiNs, whose naïve odor responses are typically weak (Aso et al., 2023). In our model, when an odor is given, excitatory signals reach KCs via ORNs and PNs, then KCs generate spikes. Here, each individual odor elicits spikes in small, distinct population of KCs. A reward stimulus given following the odor weakens the weights of KC>MBON-α1 synapses whose presynaptic KCs had been firing within 5 seconds prior to the stimulus. Although the magnitude of the decrease in the synaptic weight after a single learning is not clear, we set the initial value of *w* to 1 and the weakened value to 0.25. Reward stimuli are transmitted to KC>MBON synapses through dopaminergic neurons innervating the mushroom body (Aso et al., 2014b); however, this pathway was not modeled in this study. Whereas MBON-α1 fires in response to all odors before learning, after learning, it will reduce responsiveness only to the learned odor because the synaptic connections from the KCs representing the learned odors will be selectively weakened. Since MBON-α1 is inhibitory, the activity of SMP354, receiving input from MBON-α1 will be disinhibited and thus fire only in response to the learned odor. This activity of SMP354 represents the output of the network.

**Figure 3:**
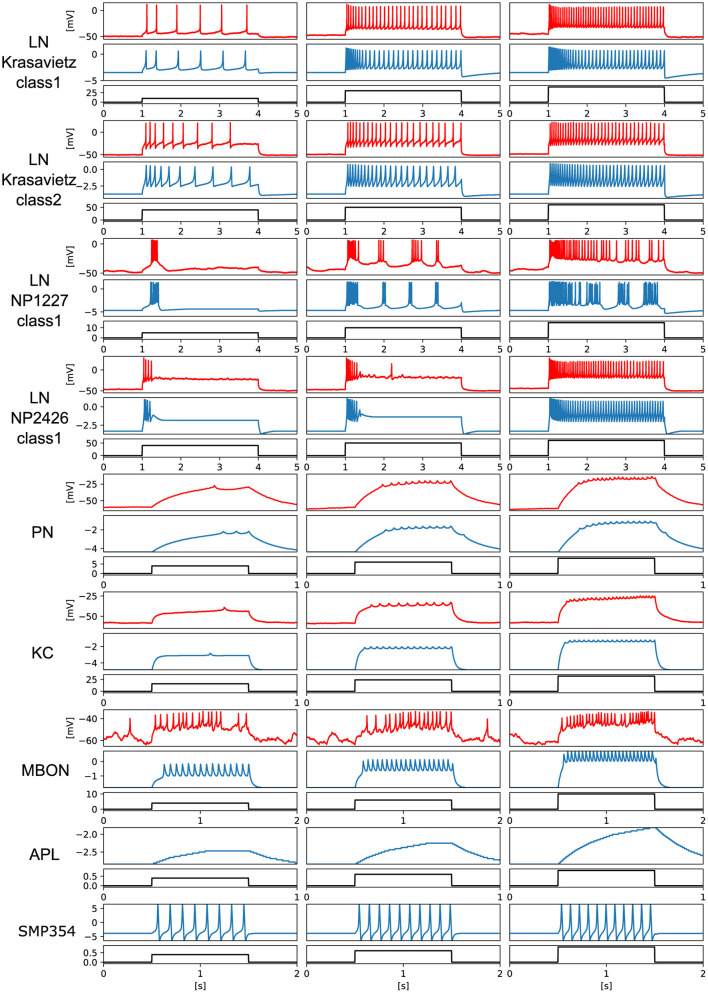
Electrophysiological properties of somatic membrane potentials of the *in vivo* data (red) and the simulated results of the PQN models *in silico* (blue) in response to step stimulus inputs (black). We conducted recordings from PNs and MBONs in this study. The data of the KC and four subclasses of the LNs are from previous studies (Seki et al., 2010; Inada et al., 2017). As there is no recorded data of APL and SMP354 neuron, we only show the simulation results.

We prepared electrophysiological data of each neuron and tuned the PQN models to replicate them. For LNs and KC, data recorded in previous studies (Seki et al., 2010; Inada et al., 2017) were used. The detailed procedures for the data acquisition from PNs and MBONs are described in the Methods section. Owing to the lack of data on APL and SMP354 neurons, only the modeling results are shown. Since we did not have data on MBON-α3, we used the one on MBON-α1. The PQN model was used to model the neurons. The parameter sets of the PQN model are shown in Supplementary Tables S1–S9. Figure 3 illustrates the responses of the somatic membrane potentials *in vivo* (red) and those of the PQN models on FPGA (blue). The black plots are the step input currents, whose unit in the recording is pA. The FPGA simulation results have no physical unit. Although a variety of LN subclasses were observed (Chou et al., 2010; Seki et al., 2010), we employed four electrophysiologically identified subclasses reported in Seki et al. (2010). They are Krasavietz_class1, Krasavietz_class2, NP1227_class1, and NP2426_class1; we fitted PQN models to each of them. The parameters of the PQN model were automatically determined using a fitting method (Nanami et al., 2017, 2018) based on the differential evolution algorithm (Storn and Price, 1997). Detailed activities of each neuron are shown in Supplementary Figures S1–S7.

### 2.2 Electrophysiological measurements

#### 2.2.1 Recording from PNs

Whole-cell patch-clamp recordings from PN somata were performed as previously described (Inada et al., 2017). Briefly, the brain of *w;UAS-ReaChR::Citrine(attP40) /+;VT033006-Gal4(attP2)/+* female flies (von Philipsborn et al., 2011; Inagaki et al., 2013), 3 days post eclosion, was removed from the head capsule and fixed on a glass slide with surgical glue (GLUture, Abbott). Part of the perineural sheath covering the antennal lobe was removed to obtain an access to cell bodies. The external saline added on top of the plate was circulated throughout the experiment. A patch pipette was pulled from a thin-wall glass capillary (1.5 mm o.d./ 1.12 mm i.d., TW150F-3, World Precision Instruments). Resistance of the pipette was typically 8–10 MΩ. The internal solution contained (in mM) 140 KOH, 140 aspartic acid, 10 HEPES, 1 EGTA, 4 MgATP, 0.5 Na3GTP, 1 KCl, and 13 biocytin hydrazide (pH ~7.2, osmolarity adjusted to ~265 mOsm). Electrophysiological recordings were made with a Multiclamp 700B amplifier (Molecular Devices) equipped with a CV-7B headstage. Signals were low-pass filtered at 2 kHz and digitized at 10 kHz. Multiple levels of depolarizing currents were injected into the soma of individual PNs to examine the relationship between the input current and the membrane potential or spike output. PNs were identified based on the signals from Citrine as well as biocytin included in the internal solution.

#### 2.2.2 Recording from MBONs

*In vivo* whole-cell current-clamp recordings from MBON-α1 and optogenetic trainings were performed as previously described (Hige et al., 2015; Aso et al., 2023). Female flies with the genotype of *10xUAS-ChrimsonR-mVenus (attP18)/w; R71C03-LexA (attP40)/LexAop-GFP (attp5); MB043C/+* reared on conventional cornmeal-based food were collected on the day of eclosion, transferred to all-trans-retinal food (0.5 mM) and kept in the dark for 48–72 h until experiments. The patch pipettes were pulled for a resistance of 4–6 MΩ and filled with pipette solution containing (in mM): L-potassium aspartate, 140; HEPES, 10; EGTA, 1.1; CaCl2, 0.1; Mg-ATP, 4; Na-GTP, 0.5 with pH adjusted to 7.3 with KOH (265 mOsm). The preparation was continuously perfused with saline containing (in mM): NaCl, 103; KCl, 3; CaCl2, 1.5; MgCl2, 4; NaHCO3, 26; N-tris (hydroxymethyl) methyl-2-aminoethane-sulfonic acid, 5; NaH2PO4, 1; trehalose, 10; glucose, 10 (pH 7.3 when bubbled with 95% O2 and 5% CO2, 275 mOsm). Whole-cell recordings were made using the Axon MultiClamp 700B amplifier (Molecular Devices). MBON-α1 was visually targeted by the GFP signal with a 60X water-immersion objective attached to an upright microscope. Cells were held at around −60 mV by injecting hyperpolarizing current. Signals were low-pass filtered at 5 kHz and digitized at 10 kHz. Data acquisition and analyses were done by custom scripts in MATLAB (MathWorks). 3-octanol (OCT) and 4-methylcyclohexanol (MCH) were presented to flies with custom odor delivery system after diluting to 1% of the saturated vapors. After recording baseline responses by alternately presenting OCT and MCH five times (duration, 1 s; interval, 30 s), OCT was paired with 625 nm LED photostimulation (pulse duration, 1 s; frequency, 0.5 Hz; power, 17 mW/mm^2^) for 1 min. MCH was presented without photostimulation for 1 min. 1.5 min later, post-pairing responses to both odors were recorded five times. The pairing resulted in selective depression of OCT responses, which is consistent with a previous study (Aso et al., 2023). The I-V relationship was measured before pairing by injecting 1-s square pulses with incrementing amplitudes (0–10 pA, 2 pA steps).

### 2.3 ORN input data

The input data were generated using the DoOR dataset (D.Münch and Galizia, 2016), which comprehensively reports the response properties of ORs of *Drosophila*. The dataset shows the response intensities of each OR for a wide variety of odorants. Given a certain odor, the firing frequency *r* of an ORN that expresses a certain OR is given by Equation (1).


(1) $$\begin{array}{l} r=c_{0}k_{j}r_{ij}+r_{spo}, \end{array}$$

where *r*_*ij*_ is the response intensity of the *i*th OR to the *j*th odorant, and its value ranges from 0 to 1. *r*_*spo*_ represents the spontaneous firing frequency, which was set to 8 from the average value examined in de Bruyne et al. (1999). *k*_*j*_ is a constant that abstractly refers to the concentration of the *j*th odorant; its values range from 0 to 1 and are listed in Supplementary Table S10. As ORNs fire at approximately 200 Hz in response to the most favorable odorants (Hallem and Carlson, 2006), parameter *c* was set to 192, such that the maximum firing frequency *r* would be 200 when *r*_*ij*_ and *k*_*j*_ were 1. Based on the Poisson process, each ORN generates a spike with probability *rdt* at every time step, where time step *dt* is 1 ms. In the input dataset, six odorants were applied sequentially for one second every five seconds. The synaptic currents from ORNs were calculated using the following Equations (2, 3).


(2) $$\begin{array}{ll} s\leftarrow 1 & \left(x=1\right) \end{array}$$

(3) $$\begin{array}{ll} \frac{ds}{dt}=-\beta s & \left(x=0\right) \end{array}$$

where *x* represents the spiking information of an ORN. *x* is 1 when a spike is emitted in the current time step by an ORN and 0 otherwise. ORNs are cholinergic (Kazama and Wilson, 2008), and their β was set to 203.125 as well as the other synapses.

We prepared three types of input data for the *in-silico* experiments. In the first type of the input data, one of the six odorants, 3-octanol, cis-3-hexenol, cyclohexanone, 2,3-butanedione, 2-hexanol, and ethyl butyrate, was applied in turn for 1 second every 5 seconds. In the second type of data, 3-octanol, was applied for ten seconds every twenty seconds. In the third type of data, the same six odorants as the first type were applied in turn for ten seconds every twenty seconds. In all *in-silico* experiments, the first type of data was initially given for 300 seconds, during which time the PN's homeostasis was adjusted (details are described in Supplementary Note 1). Subsequently, the first type of data was continuously provided, and experiments on associative learning and the activity of MBON-α1 were conducted. In contrast, in the experiments on the oscillations in the antennal lobe, the second type of data was applied following the 300-second homeostatic period. The third type of data was only used in the experiment to show the variations in oscillations for each odor (Supplementary Note 2).

### 2.4 PQN model

The piecewise quadratic neuron (PQN) model (Nanami and Kohno, 2016a,b, 2023; Nanami et al., 2016, 2017, 2018) is a qualitative neuron model designed to replicate a wide variety of neurons in the nervous system and to be efficiently implemented on digital arithmetic circuits. Compared with other qualitative models (FitzHugh, 1961; Nagumo et al., 1962; Hindmarsh and Rose, 1984), the PQN model possesses additional parameters, enabling it to represent more functional forms and reproduce a variety of neurons, each with its unique dynamical structure. In addition, although other qualitative models have cubed variable terms, which consume a vast amount of circuit resources in digital arithmetic circuits, the PQN model uses piecewise functions composed of a squared term to represent comparable dynamics and consumes few circuit resources.

The nervous system of *Drosophila* primarily comprises unipolar neurons, the soma of which is separated from the rest of the cell by a long and thin membrane. In the patch-clamp recording from the soma, only action potentials with extremely small amplitudes were observed. This is attributed to the fact that the action potentials are generated in the axon and propagated with decay to the cell body (Gouwens and Wilson, 2009). Therefore, we modeled PNs, KCs, and MBONs using two-compartment models; one compartment corresponded to the soma, and the other contained axons and dendrites. In contrast, in the soma of LNs, sufficiently large action potentials were observed (Seki et al., 2010); therefore, they were modeled using single-compartment models. APL is an inhibitory, non-spiking neuron whose axons extend to the whole mushroom body. It was reported (Inada et al., 2017; Amin et al., 2020) that APL performs local inhibition; however, the details are not clear. Therefore, in this study, we modeled APL as a simple non-spiking neuron with a single-compartment.

The equations of the PQN model in the single-compartment version for LNs, APL, and SMP354 are given by Equations (4–16).


(4) $$\begin{array}{l} \frac{dv}{dt}=\frac{\phi }{\tau }\left(f\left(v\right)-n-q+I_{b0}+m\left(I\right)\right), \end{array}$$

(5) $$\begin{array}{l} \frac{dn}{dt}=\frac{1}{\tau }\left(g\left(v\right)-n\right), \end{array}$$

(6) $$\begin{array}{l} \frac{dq}{dt}=\frac{\epsilon }{\tau }\left(h\left(v\right)-q\right), \end{array}$$

(7) $$f(v)=\{\begin{array}{l} a_{fn}{\left(v-b_{fn}\right)}^{2}+c_{fn}\;(v<0)\\ a_{fp}{\left(v-b_{fp}\right)}^{2}+c_{fp}\;(v\geq 0), \end{array}$$

(8) $$g(v)=\{\begin{array}{l} a_{gn}{\left(v-b_{gn}\right)}^{2}+c_{gn}\;(v<r_{g})\\ a_{gp}{\left(v-b_{gp}\right)}^{2}+c_{gp}\;(v\geq r_{g}), \end{array}$$

(9) $$h(v)=\{\begin{array}{l} a_{hn}{\left(v-b_{hn}\right)}^{2}+c_{hn}\;(v<r_{h})\\ a_{hp}{\left(v-b_{hp}\right)}^{2}+c_{hp}\;(v\geq r_{h}), \end{array}$$

(10) $$m(I)=\{\begin{array}{l} k_{I}m_{0}\;(I<m_{0})\\ k_{I}I\;(m_{0}\leq I\leq m_{1})\\ k_{I}m_{1}\;(I>m_{1}), \end{array}$$

(11) $$\begin{array}{l} b_{fp}=\frac{a_{fn}b_{fn}}{a_{fp}}, \end{array}$$

(12) $$\begin{array}{l} c_{fp}=a_{fn}b_{fn}^{2}+c_{fn}-a_{fp}b_{fp}^{2}, \end{array}$$

(13) $$\begin{array}{l} b_{gp}=r_{g}-\frac{a_{gn}\left(r_{g}-b_{gn}\right)}{a_{gp}}, \end{array}$$

(14) $$\begin{array}{l} c_{gp}=a_{gn}{\left(r_{g}-b_{gn}\right)}^{2}+c_{gn}-a_{gp}{\left(r_{g}-b_{gp}\right)}^{2}, \end{array}$$

(15) $$\begin{array}{l} b_{hp}=r_{h}-\frac{a_{hn}\left(r_{h}-b_{hn}\right)}{a_{hp}}, \end{array}$$

(16) $$\begin{array}{l} c_{hp}=a_{hn}{\left(r_{h}-b_{hn}\right)}^{2}+c_{hn}-a_{hp}{\left(r_{h}-b_{hp}\right)}^{2}, \end{array}$$

where *v*, *n*, and *q* correspond to the membrane potential, recovery variable, and slow variable, respectively. Parameter *I*_*b*0_ is a bias constant. Parameter *I* represents the stimulus current. The function *m* performs a nonlinear transformation of *I*, adjusting the scale of *I* with parameter *k*_*I*_ and extending the dynamic range with parameters *m*_0_ and *m*_1_. Synaptic currents from other neurons and current injections shown in Figure 2 were given to *I*. The parameters τ, ϕ, and ϵ determine the time constants of the variables. The parameters *r*_*g*_, *r*_*h*_, *a*_*x*_, *b*_*x*_, and *c*_*x*_, where *x* is *fn*, *fp*, *gn*, *gp*, *hn*, or *hp*, are constants that determine the nullclines of the variables. The parameters *b*_*fp*_, *c*_*fp*_, *b*_*gp*_, *c*_*gp*_, *b*_*hp*_, and *c*_*hp*_ are determined by other parameters such that the nullclines are continuous and smooth. All variables and parameters are purely abstract with no physical units. The initial values of all state variables were set to zero.

The equations of the two-compartment version for KCs and MBONs are given by Equations (17–20).


(17) $$\begin{array}{l} \frac{dv}{dt}=\frac{\phi }{\tau }\left(f\left(v\right)-n+I_{b0}+k_{I}m\left(I\right)-I_{c}\right), \end{array}$$

(18) $$\begin{array}{l} \frac{dn}{dt}=\frac{1}{\tau }\left(g\left(v\right)-n\right), \end{array}$$

(19) $$\begin{array}{l} \frac{dv_{s}}{dt}=\frac{\theta }{\tau }\left(-\alpha v_{s}+I_{b1}+I_{c}+k_{r}I_{r}\right), \end{array}$$

(20) $$\begin{array}{l} I_{c}=k_{0}\left(v-v_{s}\right), \end{array}$$

where *v* and *n* are the membrane potential and recovery variables in the axonal compartment, respectively, and *v*_*s*_ is the membrane potential of the somatic compartment. Parameters θ, α, and *I*_*b*1_ are the time constant, bias constant, and leakage constant, respectively. *I*_*c*_ represents the internal current that flows from the axonal compartment to the somatic compartment, and *k*_0_ is its kinetic parameter. When synaptic currents are given to *I*, the current injected into the soma (Figure 2) is given to *I*_*r*_, and *k*_*r*_ is its scaling parameter.

In PNs, homeostatic control of synaptic efficacy has been indicated (Kazama and Wilson, 2008). Although various types of homeostatic mechanisms are found in neurons, we modified the equation for PNs based on the mechanism of synaptic scaling proposed in a previous study (Turrigiano, 1999), where the weights of synaptic connections were gradually scaled according to the activity level of the postsynaptic neuron. The equations used are as Equations (21, 22).


(21) $$\begin{array}{l} \frac{dv}{dt}=\frac{\phi }{\tau }\left(f\left(v\right)-n+I_{b0}+uk_{I}m\left(I\right)-I_{c}\right), \end{array}$$

(22) $$\begin{array}{l} \frac{du}{dt}=\frac{\kappa }{\tau }\left(F_{t}-F\right), \end{array}$$

where *F* and *F*_*t*_ represent the neuronal current firing frequency and target firing frequency, respectively. The equation of *I*_*c*_ and the differential equations of *n* and *v*_*s*_ are the same as those in KCs and MBONs (Equations 18, 20). The parameter κ determines the time constant. Note that the value of *u* is fixed between 0 and 1.

The synaptic current is calculated as Equation (23).


(23) $$\frac{ds}{dt}=\{\begin{array}{ll} \alpha (1-s) & (v\geq 0),\\ -\beta s & (v<0), \end{array}$$

where *s* denotes the synaptic current and the parameters α and β determine the time constants. This synaptic model is a qualitative version of the simplified kinetic model of chemical synapses (Li et al., 2012). Although the dynamics of each synapse are unclear, the decay constants of cholinergic synapses from PN to KC and GABAergic synapses in cultured embryonic neurons have been investigated (Lee et al., 2003; Gu and O'Dowd, 2006) and are both approximately 5 ms. Therefore, the values of β were set to 203.125 so that their decay time constants were close to 5 ms. The value of α was chosen to be 250 so that a single spike results in a synaptic current amplitude of approximately 1.

The synaptic current *I* of the *i*−th neuron is calculated as Equation (24).


(24) $$\begin{array}{l} I_{i}=\overset{N}{\underset{j=1}{\sum }}w_{ji}s_{j}p_{x\text{\_}y}, \end{array}$$

where *j* represents the index of a presynaptic neuron. *w*_*ji*_ is the weight of the synaptic connection from the *j*-th neuron to the *i*-th neuron. *N* is the total number of neurons. *x* and *y* indicate the classes of presynaptic and postsynaptic neurons, respectively, and the parameter *p*_*x*_*y*_ scales the synaptic current. As the extent to which the single spike of each class of neurons affects the membrane potential of different classes of postsynaptic neurons is not known clearly, the values of *p*_*x*_*y*_ were manually fitted such that the simulation results reproduce the experimental results as closely as possible. Here, the four LN subclasses share the same *p*_*x*_*y*_ value. First, the values of *p*_*x*_*y*_ where *y* is a PN or LN were set to reproduce the characteristics of oscillations observed in the antennal lobe *in vivo*. Next, the values of *p*_*x*_*y*_, where *y* is a KC, APL, or MBON-α1, were determined to make the responses of MBON-α1 as consistent as possible with the *in vivo* data. Finally, the values of *p*_*x*_*y*_, where *y* is MBON-α3 or SMP354 neuron, are set such that the success rate of olfactory associative learning becomes as high as possible. Note that, *p*_*x*_*y*_ is positive or negative when *x* is an excitatory or inhibitory neuron. All the parameter sets of neurons and synapses are listed in Supplementary Tables S1–S9.

### 2.5 FPGA implementation of the PQN model

**Figure 4:**
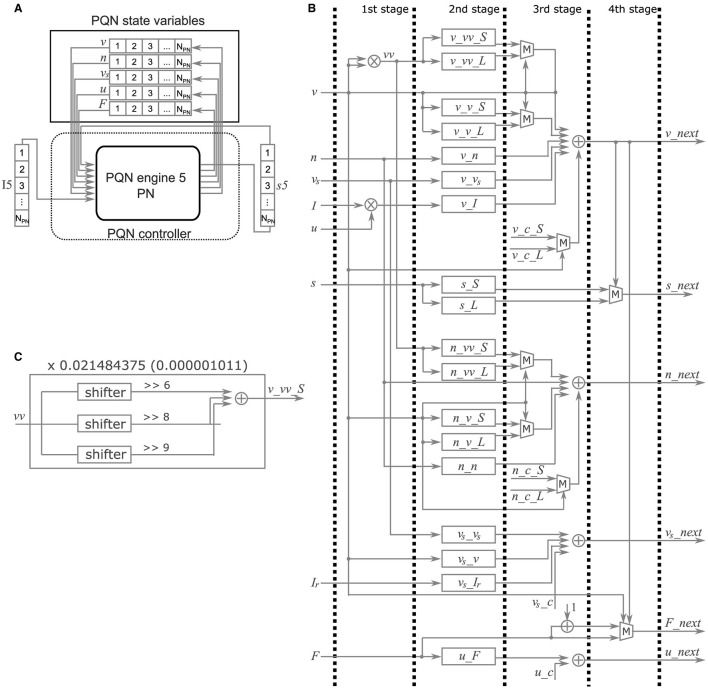
Details of the PQN engine of the PN mode. **(A)** Information flow of the PQN engine. N_PN_ is the number of PNs. **(B)** Block diagram. This circuit calculates the succeeding values of internal variables (*v*, *n*, *v*_*s*_, *u*, and *F*). The symbols × , +, and *M* represent multipliers, adders, and multiplexers, respectively. **(C)** Internal circuit for the calculation of *v*_*vv*_*S*.

In the FPGA implementation, the PQN model is simulated by the PQN engine. As an example, the details of the PQN engine of the PN mode are shown in Figure 4. Figure 4A shows the information flow of the PQN engine. The PQN engine updates the internal state variables and synaptic currents of 121 individual PNs in turn at each time step. The internal variables(*v*, *n*, *v*_*s*_, *u*, and *F*), input currents *I*, and synaptic currents *s* are sent via the PQN controller from block RAMs named PQN internal variables, I5, and *s*5 shown in Figure 6, respectively. The next step values of the internal variables and synaptic currents computed by the PQN engine are returned to the block RAMs and stored. Figure 4B shows a block diagram of the PQN engine of the PN mode. The symbols × , +, and *M* in the figure represent the multipliers, adders, and multiplexers, respectively. Each state variable is computed in four pipelined stages. In the first stage, the square of *v* and the product of *u* and *I* are calculated using two multipliers. The second stage involves multiplication of the variables and coefficients determined from the parameters, and *v*_*x*, *s*_*S*, and *s*_*L* represent the results of the calculations, where *x* is *vv*_*S*, *vv*_*L*, *v*_*S*, *v*_*L*, *n*, *v*_*s*_, or *I*. For example, the calculation of *v*_*vv*_*S* is performed by multiplying the square of *v* by 0.021484375, the binary representation of which is 0.000001011. Therefore, the calculation of the sum of the sixth, eighth, and ninth right-shift operations on the square of *v* is performed (Figure 4C). In the third stage, the values of *v*, *n*, *v*_*s*_, and *u* are calculated. In the fourth stage, the values of *s* and *F* are determined based on the new value of *v*. When the old value of *v* is negative and the new value is zero or greater, the spike detector detects a spike. The values of *v*, *n*, *v*_*s*_, and *F* are updated every 1 ms, whereas the value of *u* is updated only once per second. The current firing frequency is calculated from the number of spikes counted in one second. All state variables are expressed in an 18-bit fixed-point representation, of which 10 bits are the decimal part and the remaining are the integer part.

## 3 Results

### 3.1 Olfactory associative learning

**Figure 5:**
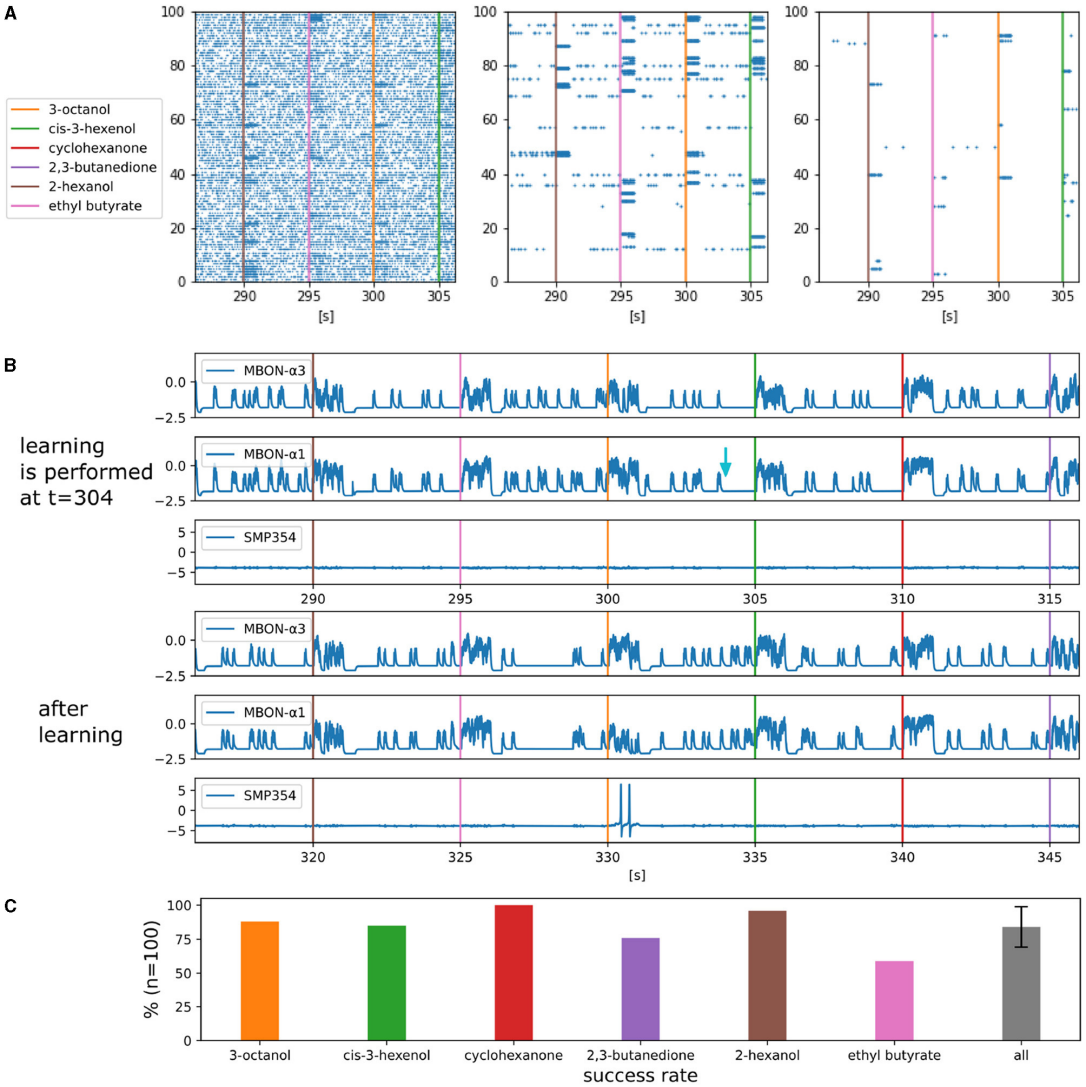
Activities of each type of neuron and the success rate of olfactory associative learning. **(A)** Parts of the raster plot of ORNs, PNs, and KCs. The horizontal axis represents time and the vertical axes represents indices of the neurons, respectively. The colored bars represent the onset of the one-second odorant applications. The blue dots represent spikes. **(B)** Waveforms of somatic membrane potentials of MBON-α3, MBON-α1 and SMP354 neurons during and after learning. The light blue arrow indicates the timing of the reward signal that triggered learning. **(C)** Success rates of olfactory associative learning for six odorants. Error bars represent standard deviation over the success rates of the six odorants.

Flies are capable of olfactory associative learning, where they remember the odor associated with the reward. One of the main goals of our model is to reproduce the neuronal mechanisms underlying this learning. Figure 5A shows a portion of the raster plots for ORNs, PNs, and KCs. Every 5 seconds, one of the six odorants, 3-octanol, cis-3-hexenol, cyclohexanone, 2,3-butanedione, 2-hexanol, and ethyl butyrate, was applied in turn for one second. As the responses propagate from ORNs to PNs to KCs, a smaller number of neurons are activated. These have also been observed in the olfactory nervous systems in multiple species (Wilson et al., 2004; Turner et al., 2008). This sparse activity of KCs suggests that individual odors are represented by a small number of KCs, which in turn allows flies to selectively identify the odor associated with the reward.

Figure 5B shows the activities of MBONs and SMP354 neuron before and after olfactory associative learning *in silico* (FPGA). The application of 3-octanol was followed by a reward signal at *t* = 304. This resulted in LTD at KC>MBON-α1 synapses, the presynaptic neurons of which fired in the previous five seconds. Subsequently, MBON-α1 became selectively unresponsive to 3-octanol, whereas MBON-α3 remained responsive to all odorants. Consequently, SMP354 neuron that receives excitatory input from MBON-α3 and inhibitory input from MBON-α1 fires only when 3-octanol is applied.

Figure 5C shows the success rates of olfactory associative learning for individual odors. Each set of experiments comprised one associative learning and ten trials. In each trial, all six odors were applied sequentially in a unique order. A trial was considered successful when SMP354 neuron responded solely to the learned odor. Ten sets of experiments comprising 100 trials were conducted for each odor, and the probabilities of success were calculated. This model achieved an average success rate of 84.0%. The variation in the results of each trial originates from the variable input spike streams from ORNs.

### 3.2 Oscillations in the antennal lobe

**Figure 6:**
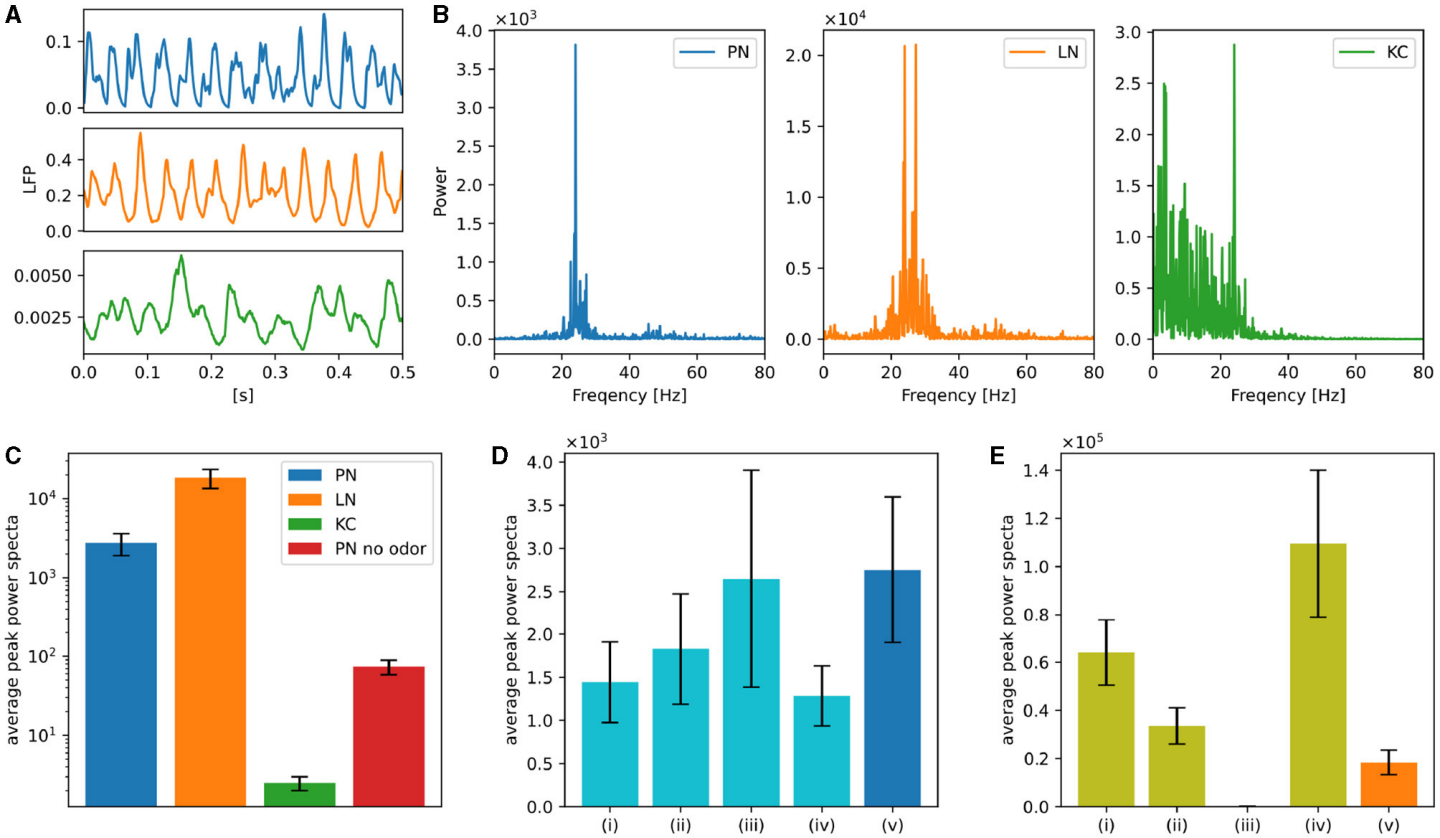
Neuronal oscillations in PNs, LNs, and KCs. **(A)** Examples of the virtual LFP for each type of neuron. **(B)** Power spectra of the virtual LFPs of PNs, LNs, and KCs. 3-octanol was applied for ten seconds. **(C)** Averages of the peak power spectra were plotted on a logarithmic scale graph. Average of the peak power spectra of PNs in the absence of the odor was also plotted. Error bars represent standard deviation over 20 trials. **(D)** Averages of the peak power spectra when one of the four types of LNs was inactivated. LNs of Krasavietz_class1 (i), Krasavietz_class2 (ii), NP1227_class1 (iii), and NP2426_class1 (iv) were inactivated, respectively. The results obtained without inactivation are also plotted for comparison (v). Error bars represent standard deviation over 20 trials. **(E)** Averages of the peak power spectra of each LN subclasses. Krasavietz_class1 (i), Krasavietz_class2 (ii), NP1227_class1 (iii), and NP2426_class1 (iv). The average peak power spectra of the virtual LFPs over all LNs were also plotted for comparison (v). Error bars represent standard deviation over 20 trials.

In order to test whether our model is applicable to known activity dynamics observed in the *Drosophila* olfactory system other than plastic changes induced by learning, we next focused on neuronal oscillations. Neuronal oscillations are widely observed in the olfactory nervous system of insects and are believed to be important in odor information processing (Stopfer et al., 1997; Perez-Orive et al., 2002). Oscillations have also been reported (Tanaka et al., 2009) in the PNs of *Drosophila*, which are absent without odors or when LNs are inactivated. A similar oscillatory behavior was observed in our model. Whereas Tanaka et al. (2009) measured the local field potential (LFP) caused by the synaptic currents of PNs, we calculated a virtual LFP by averaging the synaptic currents for each type of neuron. Figure 6A shows the virtual LFP of PNs, LNs, and KCs when 3-octanol was applied, where clear oscillations can be seen in PNs and LNs. The peak amplitudes of their frequency spectra were estimated to clarify their oscillatory nature. Figure 6B shows the power spectra (details are explained in Supplementary Note 3), which have the peak at approximately 20–30 Hz. Here, the odor was applied for ten seconds. Following this, we applied 3-octanol twenty times and plotted the averaged values of the peak power on a logarithmic scale (Figure 6C). The peak power of LNs was considerably higher than that of PNs; whereas, the peak power of KCs was much smaller than that of PNs, which is consistent with Turner et al. (2008) reporting no clear oscillations in the membrane potentials of the *Drosophila* KCs. In addition, the peak power when odor was not given was much lower than that under normal conditions, which is consistent with the results in Tanaka et al. (2009).

The previous study (Tanaka et al., 2009) selectively inactivated the synaptic output of NP1227_class1 and NP2426_class1 LNs in turn, and reported that the oscillations of PNs were attenuated only when NP2426_class1 was inactivated. We inactivated each subclass of LNs in turn and plotted the average of their peak spectra of the oscillations of PNs (Figure 6D). Here, inactivation of LNs was performed by forcing the stimulus input to the LNs to zero. 3-octanol was applied five times for each condition. The peak power was significantly attenuated when NP2426 class1 but not NP1227 class1 was inactivated. This is consistent with the experimental results in Tanaka et al. (2009). The second and third largest attenuation was observed following the inactivation of Krasavietz class1 and class2, respectively.

We also calculated the virtual LFP for each LN subclass under the normal condition. Figure 6E shows the average peak power when 3-octanol was applied twenty times. The peak power of NP2426_class1 was the largest, indicating that it was the primary source of oscillation in LNs. The peak powers of Krasavietz_class1 and Krasavietz_class2 are the second and third largest, respectively. There are almost no oscillations in NP1227_class1, which could explain why inactivation of NP1227_class1 does not attenuate the oscillations in PNs.

### 3.3 Temporal dynamics of firing in MBON-α1

**Figure 7:**
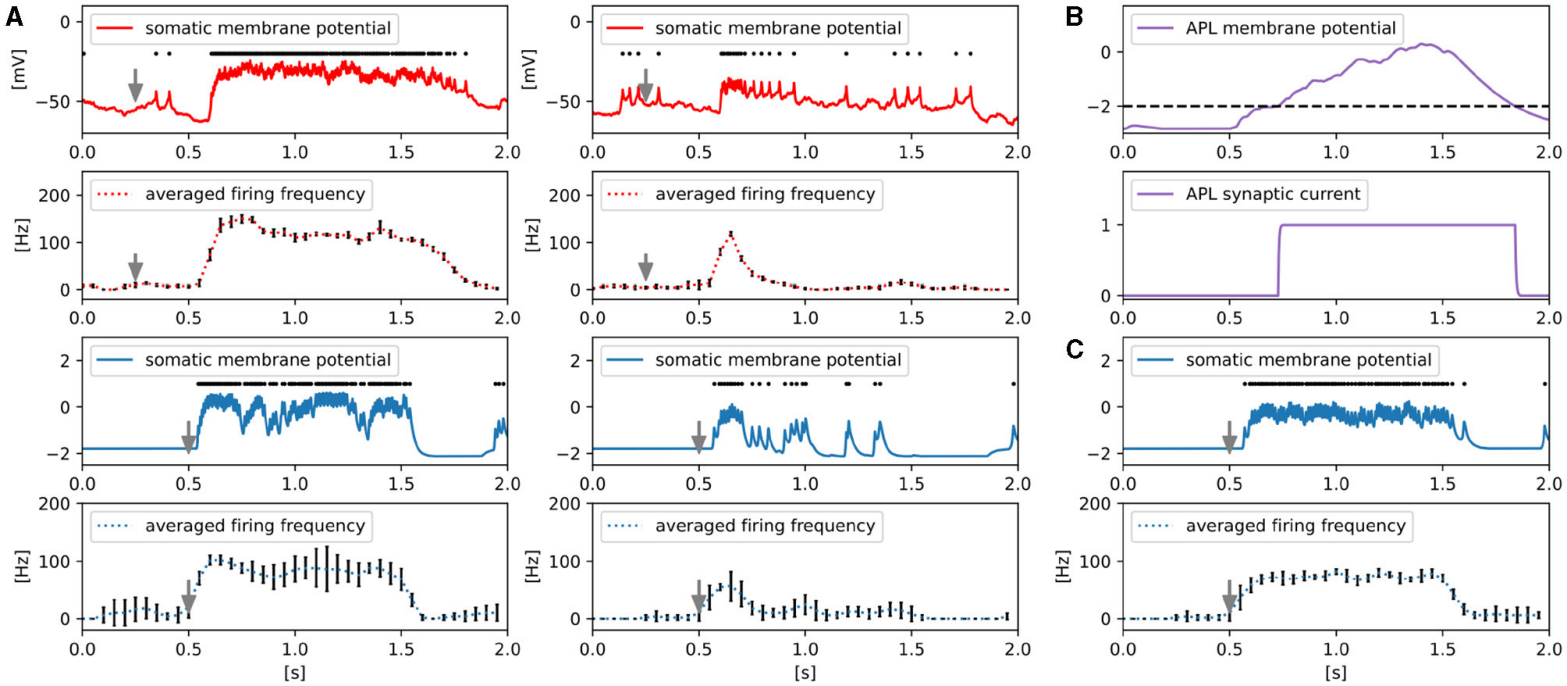
Responses of MBON-α1. **(A)** Comparison of somatic membrane potential of MBON-α1 *in vivo* (red) and *in silico* (blue) before and after olfactory associative learning. The gray arrows indicate the onset of a 1-second-long application of 3-octanol. *In silico*, the odor input caused ORNs to fire instantaneously, whereas *in vivo*, there was a delay for the odor to travel through the tubing and reach ORNs. Example responses of the somatic membrane potential for a single trial (solid line) and the temporal dynamics of firing frequency averaged over five trials dotted line). Error bars represent standard deviation over five trials. The black dots above the solid lines represent spikes. **(B)** The membrane potential and synaptic current of APL are shown. The black dotted line represents the threshold for the release of the synaptic current of APL. **(C)** Example responses of the somatic membrane potential of MBON-α1 in the absence of APL for a single trial (solid line) and its averaged firing frequency over five trials (dotted line). Error bars represent standard deviation over five trials. The black dots above the solid line represent spikes.

Figure 7A shows the responses of the somatic membrane potential of MBON-α1 *in vivo* (red) and *in silico* (blue) before and after olfactory associative learning. The solid plots represent the values of the somatic membrane potential, and the black dots above them represent the detected spike timing. The gray arrows indicate the onset of 3-octanol input. 3-octanol was given for 1 second. We calculated the firing frequency for each 50 ms time window from the spikes and plotted the average firing frequency transition over five trials as dotted curves. The procedures for detecting spikes and calculating their frequency are described in Supplementary Note 4.

*In vivo*, whereas odor-evoked firing frequency of MBON-α1 is constantly high before learning, it decreases rapidly after learning. We reproduced this characteristic temporal dynamics of firing *in silico*. The somatic membrane potential and synaptic current of APL are shown in Figure 7B to illustrate how the temporal dynamics occur *in silico*. While MBON-α1 fires immediately after odor onset, the membrane potential of APL reaches the threshold with a delay due to its slow neuronal dynamics. This delayed inhibition from APL may contribute to suppressing the firing of MBON-α1 from approximately *t* = 0.7, together with LTD at KC>MBON-α1 synapses. We tested this possibility *in silico* by examining the activity of MBON-α1 while inactivating APL (Figure 7C). Without APL, the odor-evoked firing frequency of MBON-α1 is constantly high even after learning, and this result indicates that APL is essential for the temporal activity of MBON-α1.

### 3.4 FPGA implementation

**Figure 8:**
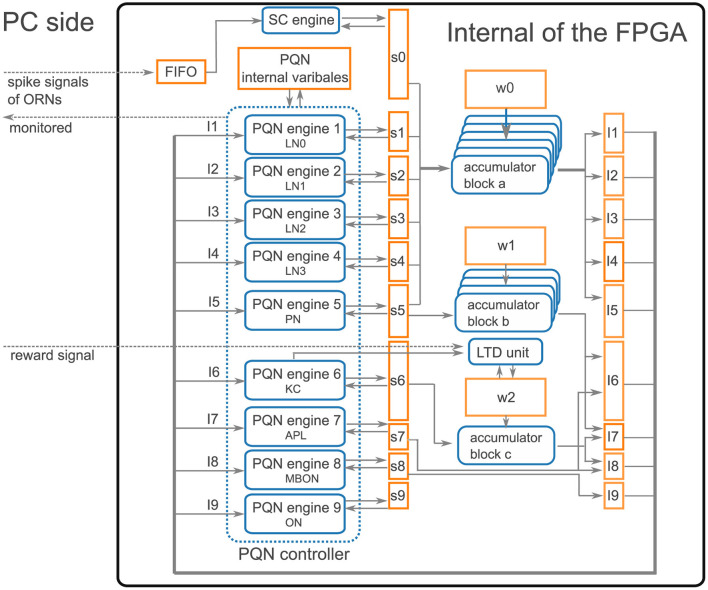
Architecture overview. The rectangle, rounded rectangle, and arrows represent the block RAM, computation unit, and data flow, respectively. PQN engines, where *x* ranges from 1 to 9, simulate the activities of each type of neuron. LN0, LN1, LN2, and LN3 correspond to Krasavietz_class1, Krasavietz_class2, NP1227_class1, and NP2426_class1, respectively. s*x*, where *x* ranges from 0 to 9, and I*y*, where *y* ranges from 1 to 9, are the synaptic and input currents, respectively. w0, w1, and w2 store the weight of synaptic connections. The PQN internal variables store neuronal internal state variables. Spike signals of ORNs are sent from the PC using serial communication. They are temporarily stored in the FIFO and subsequently converted to synaptic currents using the SC engine. The accumulator blocks a, b, and c comprise seven, three, and one accumulator(s), respectively, and each accumulator calculates the input currents from the synaptic currents in parallel. The PQN controller activates each PQN engine in turn to simulate neurons. Each PQN engine receives the internal variables, input currents, and synaptic currents of the corresponding type of neurons and returns the next step values of the internal variables and synaptic currents. The reward signal from the PC activates the LTD unit and reduces the weights of KC>MBON-α1 synapses that are stored in part of w2. The information required for each result section, such as the spike information of the PQN neurons, values of membrane potential, and synaptic currents, is selected and sent to the PC and stored.

Our qualitative modeling approach allowed to implement the entire model on an entry-level FPGA (Xilinx Artix-7 XC7A35T on a Digilent cmod-a7 board) using Xilinx Vivado 2016.4. Figure 8 presents an overview of the implementation. As the network has nine types of neurons, namely four LN subclasses, PN, KC, APL, MBON, and SMP354 neuron, we constructed nine PQN engines corresponding to each of them. The weights of synaptic connections, input current, synaptic current, and neuronal internal state variables are stored in block RAMs. Spike signals of ORNs were generated by the PC and sent to the FPGA through a serial communication bus. The spike signals were composed of 11 bits representing the indices of ORNs, which were initially stored in the FIFO buffer; the synaptic currents of ORNs were calculated using the SC engine. The accumulators calculated the input currents for neurons from the synaptic currents in parallel. The antennal lobe and the mushroom body are distant, and only PNs provide a one-way connection from the antennal lobe to the mushroom body. Therefore, we built three accumulator blocks, a, b, and c, which consisted of seven, three, and one accumulator(s), that were responsible for the processing inside the antennal lobe, between the antennal lobe and the mushroom body, and inside the mushroom body, respectively. The weights *w* of the KC>MBON-α1 synapses are represented by two bits to realize the LTD, whereas all other synaptic weights are represented by one bit. The PQN controller activates each PQN engine in turn. Each PQN engine receives the current values of the internal variables, input currents, and synaptic currents of the corresponding type of neurons. It then returns the next step values of the internal variables and synaptic currents. A reward signal was also transmitted using serial communication to the LTD unit, which triggered the LTD of KC>MBON-α1 synapses. The LTD unit holds the indices of KCs that have fired in the previous five seconds, and when the reward signal arrives, it rewrites *w* of synapses made by those KCs onto MBON-α1.

**Figure 9:**
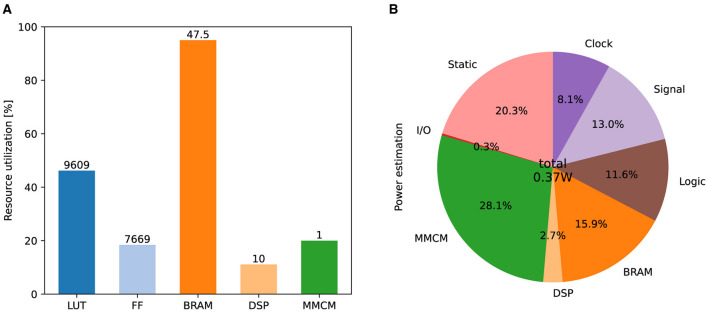
Results of FPGA implementation. **(A)** Utilization ratio for each type of resource. The numbers above the bar indicate the number of units used. **(B)** Power estimation for each type of resource.

Figure 9A shows the resource consumption of this implementation. The look-up tables (LUTs) are truth tables that were used primarily for addition calculations in this implementation. Digital signal processors (DSPs) are blocks for complex calculations that were used to multiply the state variables. Flip-flops (FFs) and block random-access memories (BRAMs) are memory elements. Most BRAMs store synaptic weights, whereas the rest store state variables. A mixed-mode clock manager (MMCM) was used to generate a 100 MHz clock. Figure 9B lists the on-chip power consumption of each resource estimated by Vivado. The static represents the steady-state leakage power of the device and is independent of the circuit design. The total power consumption is approximately 0.37 watts.

## 4 Discussion

In this study, we built the first data-driven SNN model of the olfactory nervous system of *Drosophila melanogaster*. Our modeling approach proposed a way to overcome the trade-off between replicating the detailed biological data (the connectome and electrophysiological activities of neurons) and the computational cost, such that the model can run in real-time on a low-power SiNN chip while reproducing the characteristic neuronal activities in the brain. Features of previous data-driven models (Markram et al., 2015; Bezaire et al., 2016; Ecker et al., 2020) that reproduced parts of the mammalian cortex and hippocampus as well as this work are compared in Table 1. Specifically, our model went beyond the preceding models in the following four aspects: the higher reproducibility of (1) synaptic connectivity, (2) characteristic spiking activities, (3) neuronal functions, and (4) the lower computational cost. Whereas the preceding models reproduced the electrophysiological and morphological properties of each type of neuron using multicompartmental ionic-conductance-based models, our model reproduced electrophysiological properties using the PQN model, which requires a lower computational cost. In Markram et al. (2015) and Ecker et al. (2020), the Tsodyks–Markram (TM) synapse model (Tsodyks and Markram, 1997) with a stochastic mechanism was used to accurately reproduce synaptic physiology, whereas in Bezaire et al. (2016), the double exponential synapse model reproduced the rising and decaying time constants of the synaptic current for each type of synaptic connection. In this study, the decay time constant of the double exponential synapse model was fitted to electrophysiological data for the corresponding type of neurotransmitter. In the preceding models, synaptic connections were randomly determined based on the position and morphology of individual neurons and statistical information for each neuron type. However, in this model, they were based on the connectome (HEM, 2020; Scheffer et al., 2020) identified from comprehensive electron microscopy images. In the preceding models, the vast number of neurons and complex structures of the mammalian brain limited the validation of the models. In Markram et al. (2015) and Bezaire et al. (2016), synchronous oscillations at the network level were validated, but not for each type of neuron. Spiking activities were not examined in Ecker et al. (2020). Additionally, the preceding models did not reproduce the function of the network, as mammalian cortical and hippocampal functions at the circuit level have not yet been elucidated. In contrast, because the olfactory nervous system has a smaller network size and its function is clearer, we were able to demonstrate that our model successfully reproduces olfactory associative learning, characteristic spiking activities of each type of neuron, such as odor-evoked oscillatory firing in PNs and LNs, absence of oscillations in KCs, different contributions of LN subclasses to the formation of oscillations, and temporal dynamics of firing in MBON-α1. Whereas the preceding models required supercomputers owing to their enormous computational cost, our model was light enough to be simulated on an entry-level low-cost FPGA chip at 0.37 watts, which may be acceptable for small robots and portable AI devices. In addition, whereas the simulation speed in Bezaire et al. (2016) was approximately 1,600 times slower than real time, our model performs real-time simulations.

**Table 1:** Comparison of the data-driven SNN models.

	**This paper**	**(Markram et al., 2015)**	**(Bezaire et al., 2016)**	**(Ecker et al., 2020)**
Target	*Drosophila* olfactory nervous system	Microcircuit of rat neocortex	CA1 of rat hippocampus	CA1 of rat hippocampus
Model	PQN model	Ionic-conductance -based model	Ionic-conductance -based model	Ionic-conductance -based model
Scale	2,200 neurons	31,000 neurons	340,000 neurons	400,000 neurons
Reproducibility of neuronal electrophysiology	High	High	High	High
Reproducibility of neuronal morphology	No	High	High	High
Reproducibility of synaptic properties	Low (PQN synapse)	High (TM with stochastic)	Medium (double exponential)	High (TM with stochastic)
Reproducibility of synaptic connectivities	High (connectome-based)	Medium ( morphology-based statistical method )	Medium (neuronal distance -based statistical method )	Medium ( morphology-based statistical method )
Reproducibility of characteristic spiking activities	High	Medium	Medium	Low
Reproducibility of functions	High	No	No	No
Computing environment	FPGA(0.3W)	Supercomputer (4-rack IBM Blue Gene/Q)	Supercomputer (3,488 processors)	Supercomputer
Simulation speed	Real time	Not available	1,642 times slower	Not available

There also are differences between our model and the latest preceding model (Kennedy, 2019) of the *Drosophila* olfactory system. Unlike our model, the preceding model is not data-driven. The preceding model used the leaky I&F model, and did not reproduce the electrophysiological properties of each class of neurons. As for the structure of the network, our model employs a slightly extended version of the preceding model. Whereas the preceding model consists of PNs, LNs, KCs, APL, and MBON, our model has another MBON and SMP354 neuron in addition, reproducing the valence-balance model (Heisenberg, 2003; Aso et al., 2023), where learning-induced plasticity in the KC>MBON synapses tips the balance of valence signals of MBONs. This competitive memory circuitry is important because it is the basis for the interactions among MBONs that are responsible for flexible and complex behavioral decisions associated with memory. As for the learning rule, both models employ reward-induced depression of KC>MBON synapses to implement olfactory associative learning. As for the synaptic connections, whereas the preceding model stochastically determines the connections between layers such as ORN>PN and PN>KC, our model precisely reproduces the connections based on the connectome database.

As for the spiking dynamics, the characteristic spiking activities of each neuron are not considered in the preceding model. For example, the spiking activities of PNs and LNs are not calculated by spiking neuron models but are generated by the Poisson process. The activities of MBONs are represented using nonlinear activation functions. KCs are described by the LIF model, and their firing properties are not fitted to the *in vivo* data.

The peak frequency of PN oscillation in this model was approximately 24 Hz, whereas experimentally observed peak frequency in the antennal lobe was 10–15 Hz (Tanaka et al., 2009). In the antennal lobe, PNs and LNs are connected via glomeruli, which are neuropils comprising the dendrites and axons of PNs, LNs, and ORNs. However, the model does not consider the dynamics of the glomeruli, which may cause a gap in peak frequencies. In addition, the proportion and detailed connections of the four subclasses of LNs are not known; therefore, they were not incorporated into the model and may have affected the peak frequency. A more detailed model awaits to be built to clarify the mechanism and function of oscillations in the antennal lobe.

To examine the oscillations (Figure 6), we only applied 3-octanol to the network. This is because the magnitude of PN's oscillations greatly depends on the identity of odors both *in vivo* (Tanaka et al., 2009) and *in silico* (Supplementary Figure S8). Since our intention was to measure the effect of inactivation of the LN subclass on PN's oscillations, we used only one type of odor. In the future, we will comprehensively examine the relationship between oscillations and odors and clarify why the magnitude of the oscillations differs between odors.

In honeybees, oscillations in the antennal lobe are necessary for distinguishing between similar odors (Stopfer et al., 1997). In locusts, oscillations appear not only in the antennal lobe but also in KCs, and they are believed to contribute to the sparse representation of odors in the KC population (Perez-Orive et al., 2002). Although the role of oscillations in *Drosophila* remains unclear, oscillations likely contribute to the processing of odor information given the similarity of olfactory network structure between different insects. One possible candidate is the generation of the sparse representation of odors in the antennal lobe.

In this study, the PQN model employs function *m*(*I*), which was not incorporated into the original PQN model (Nanami and Kohno, 2023). This function performs a nonlinear transformation of the stimulus input so that the membrane potential behaves as expected in response to a wide range of stimulus inputs. However, this function does slightly complicate the model and has no biological counterpart. By changing the parameters and adjusting the dynamics, we expect to be able to remove this function in future works.

As shown in Figure 7, after olfactory associative learning, MBON-α1 fires for approximately 250 ms, immediately after the arrival of the odor signal, and subsequently enters a resting period, successfully reproducing the temporal firing observed in Hige et al. (2015) in MBON-γ1pedc. To our knowledge, there has been no report on the mechanism underlying this firing dynamics characteristic for the post-learning response. The result of our simulation suggests that the delayed activation of APL contributes to shaping this activity pattern. Thus, our modeling not only reproduces observed physiological data but also provides mechanistic insight by proposing an experimentally testable hypothesis.

The SiNN implemented in this study operates at the same speed as the olfactory nervous system with a 100 MHz clock signal. However, if we use a higher clock, the model can provide accelerated simulations, albeit with increased power consumption. For example, we confirmed that the model can simulate four times faster than real-time using a 400 MHz clock with a Xilinx Virtex UltraScale+ xcvu37p-fsvh2892-3-e FPGA. In this implementation, the estimated power consumption was about 4W. The power efficiency and simulation speed can be further improved by using Application Specific Integrated Circuits (ASICs).

As shown in Figure 9B, most of the power is consumed by the MMCM, BRAMs, and the steady-state leakage (Static). Except for a few BRAMs that are used to store the neuronal state variables, these resources are not directly used to compute the neuronal dynamics. Ignoring the reproducibility of the spiking properties and using I&F-based models instead of PQN might reduce the power of clocks, signals, logic, and DSPs. However, these resources consume only 18.5% of the total power and their impact on the overall system is expected to be small. Ionic-conductance-based models can reproduce the dynamics of the spiking process as accurately as or better than the PQN model. However, they have many exponential terms that consume a large number of DSPs in FPGA implementations (Akbarzadeh-Sherbaf et al., 2018; Khoyratee et al., 2019). Even in the most well-optimized implementation (Khoyratee et al., 2019), it requires more than 20,000 LUT units and more than 100 DSPs to build a network of 2,000 neurons, which would lead to significantly higher power consumption.

Our modeling approach is applicable to not only FPGAs but also ASICs. Conversion from FPGA to ASIC improves power efficiency by a factor of 14 to 20 (Amara et al., 2006; Kuon and Rose, 2007). The network reproduced in this study accounts for approximately 2% of the entire brain. Thus, our approach enables the construction of an ASIC chip that simulates the entire *Drosophila* brain while consuming approximately 1 watt. Such chips have considerable potential in the engineering and scientific fields. Because of its low power consumption, the chip can be mounted on small insect-like robots. The resulting system is expected to move around autonomously, solve unknown tasks, and adapt to changes in the environment, similar to insects. In addition, owing to its intrinsic power efficiency, the chip can serve as a sufficiently fast simulator of the whole brain within the constraints of the power supply typically available in laboratories. It can facilitate long-term measurement of neuronal activities and is expected to contribute to the analysis of phenomena with long timescales, such as continuous learning and forgetting.

**Figure 10:**
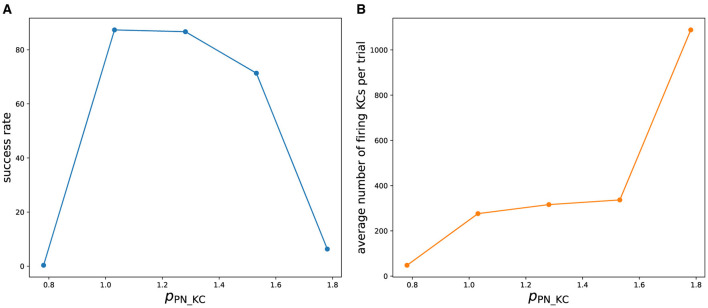
**(A)** Success rate of olfactory associative learning while changing *p*_PN_KC_. To calculate the success rate, 50 trials were performed for each odor. **(B)** Average number of firing KCs per trial.

To evaluate the robustness of our approach, we measured how the success rate of the olfactory associative learning varied while changing one of the empirically determined parameters (Figure 10A). We varied *p*_PN_KC_ which scales the strength of synaptic connections from PN to KC. Increasing or decreasing from the original value (*p*_PN_KC_ = 1.03125) decreased the success rate. This is because at the lower value, the inputs from PNs to KCs are weakened, and KCs rarely fire (Figure 10B). As a result, KC>MBON synaptic depression, which is the basis of learning, does not occur sufficiently. When *p*_PN_KC_ is large, too many KCs fire, preventing the sparse representation of odors in KCs and reducing the success rate. At present, these parameters have to be carefully tuned manually, which hinders the easy application of this approach to other nervous systems. In future research, we plan to develop a method to automatically determine these parameters to achieve the functionality of the network. Metaheuristics will be applied, just as we determined the parameters of neurons by the differential evolution algorithm.

## Data availability statement

Codes and data are deposited in GitHub https://github.com/tnanami/fly-olfactory-network-fpga/tree/main.

## Ethics statement

The manuscript presents research on animals that do not require ethical approval for their study.

## Author contributions

TN: Conceptualization, Data curation, Formal analysis, Funding acquisition, Investigation, Methodology, Project administration, Resources, Software, Validation, Visualization, Writing – original draft, Writing – review & editing. DY: Data curation, Investigation, Resources, Writing – original draft. MS: Data curation, Investigation, Resources, Writing – original draft. TH: Methodology, Writing – original draft, Writing – review & editing. HK: Methodology, Writing – original draft, Writing – review & editing. TK: Conceptualization, Methodology, Supervision, Writing – original draft, Writing – review & editing.
