# Supplementary material for: A lightweight data-driven spiking neuronal network model of Drosophila olfactory nervous system with dedicated hardware support
Source: Front Neurosci. 2024 Jun 26;18:1384336. doi: 10.3389/fnins.2024.1384336 (PMC11238178; doi:10.3389/fnins.2024.1384336)
Supplement: Supplementary file 1 [file Data_Sheet_1.pdf]

Supplementary Material

1 SUPPLEMENTARY FIGURES

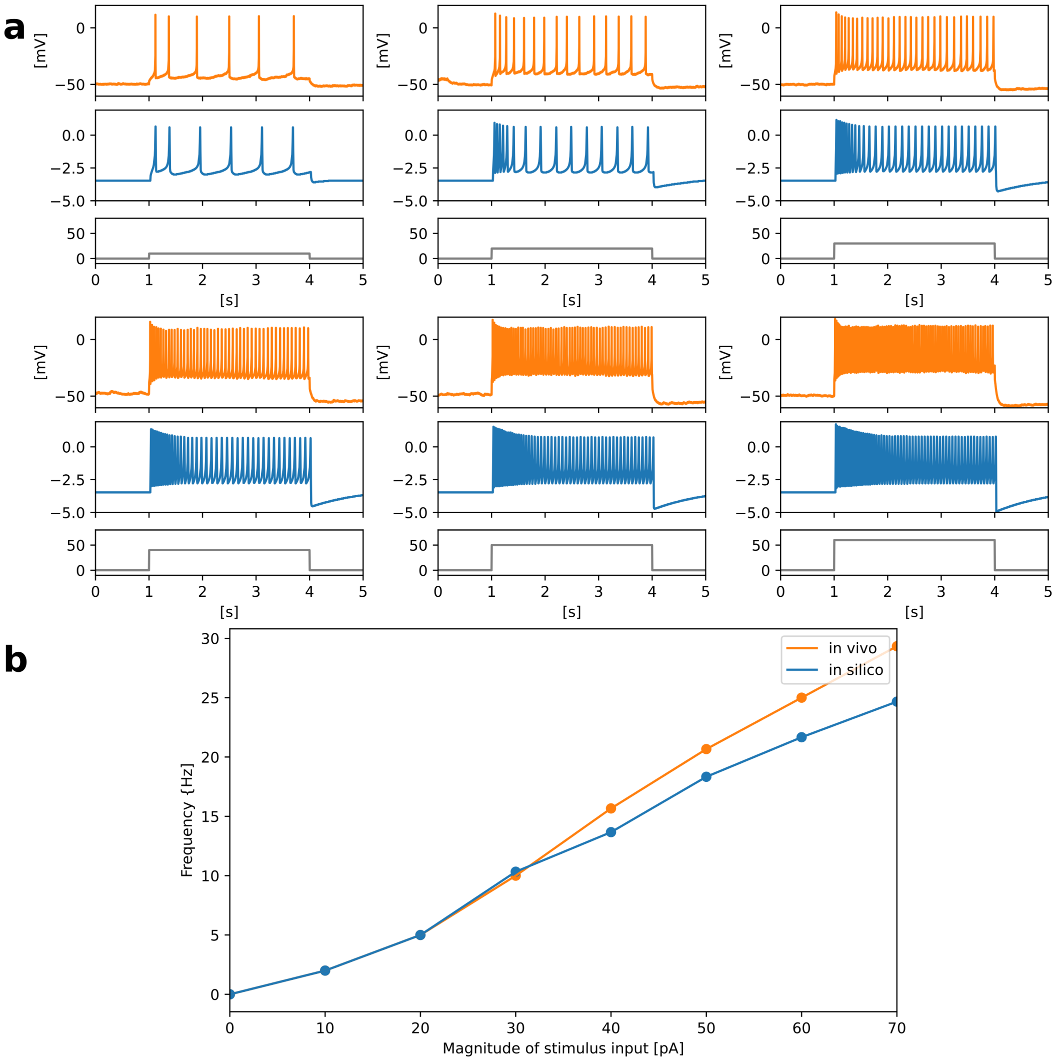

Figure S1. Responses of Krasavietz class1 in vivo and in silico. **a** Responses of somatic membrane potentials in vivo (orange) and in silico (blue) in response to step stimulus inputs of several magnitudes. **b** Transition of firing frequency. The horizontal axis represents the magnitude of stimulus input, and the vertical axis represents the frequency.

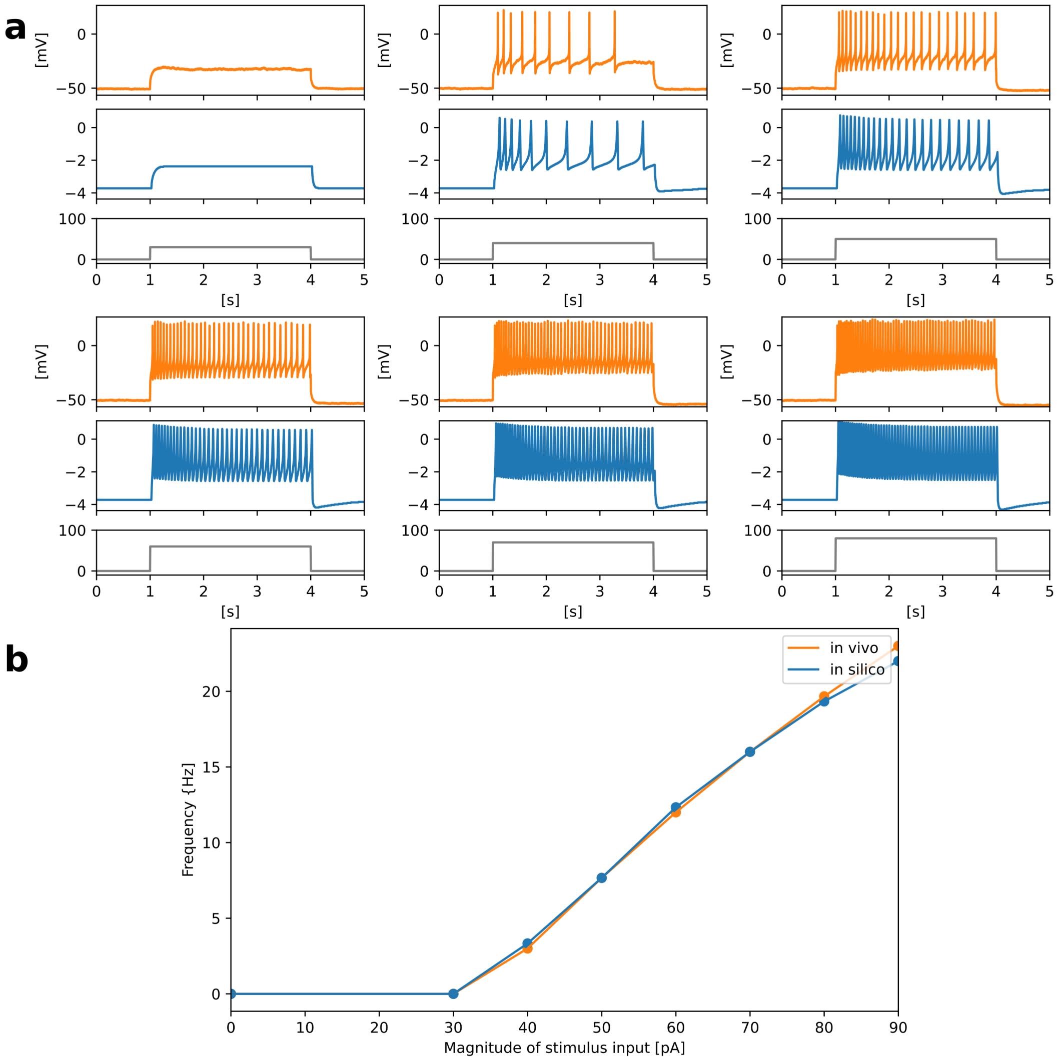

Figure S2. Responses of Krasavietz class2 in vivo and in silico. **a** Responses of somatic membrane potentials in vivo (orange) and in silico (blue) in response to step stimulus inputs of several magnitudes. **b** Transition of firing frequency. The horizontal axis represents the magnitude of stimulus input, and the vertical axis represents the frequency.

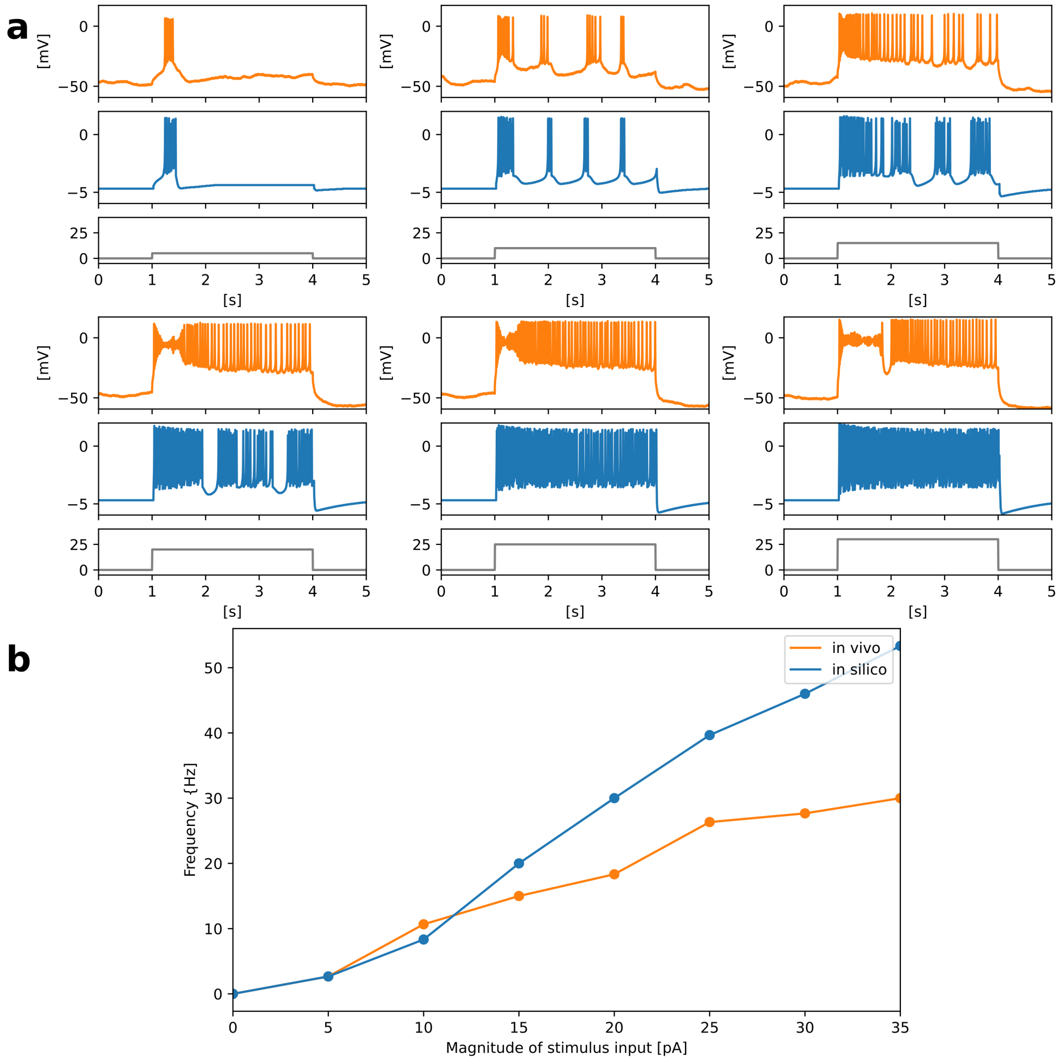

Figure S3. Responses of NP1227 class1 in vivo and in silico. **a** Responses of somatic membrane potentials in vivo (orange) and in silico (blue) in response to step stimulus inputs of several magnitudes. **b** Transition of firing frequency. The horizontal axis represents the magnitude of stimulus input, and the vertical axis represents the frequency.

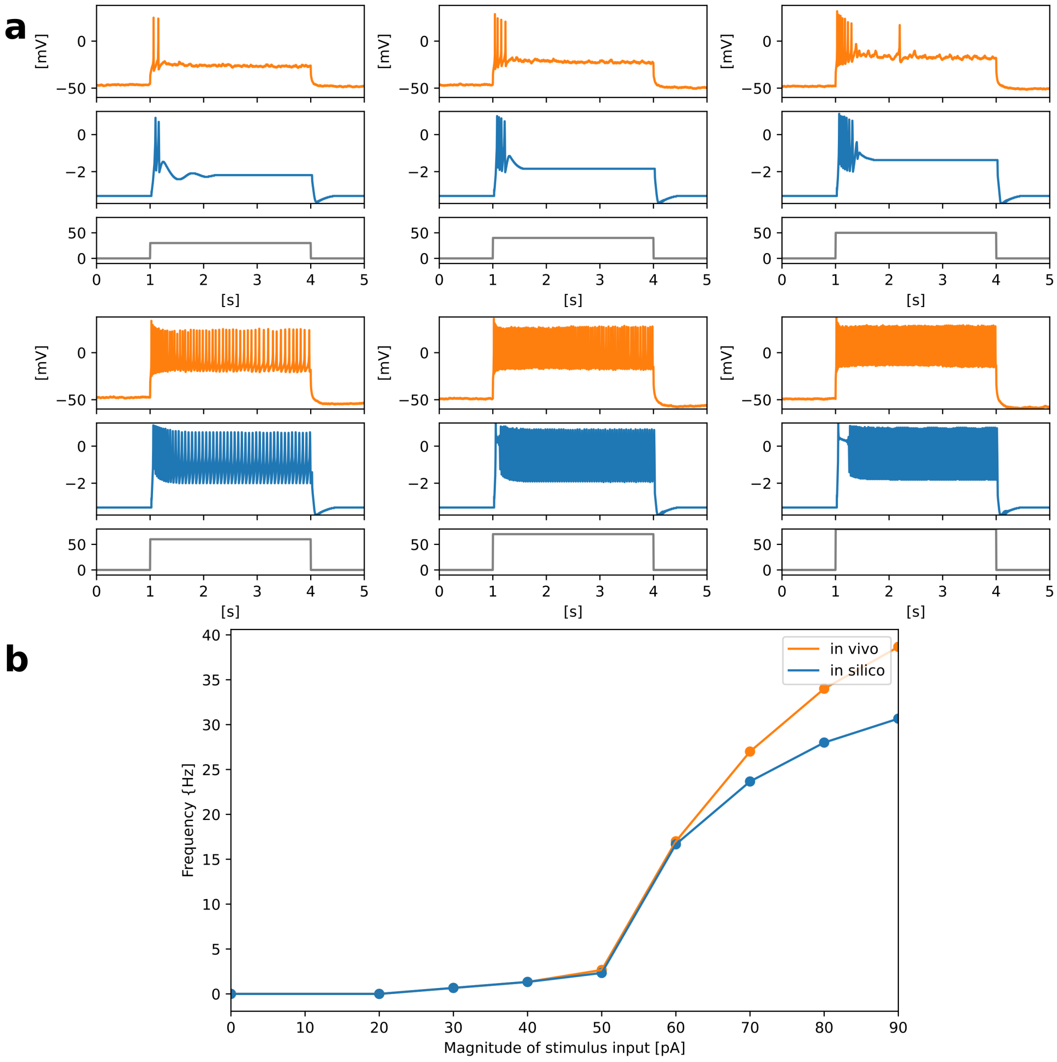

Figure S4. Responses of NP2426 class1 in vivo and in silico. **a** Responses of somatic membrane potentials in vivo (orange) and in silico (blue) in response to step stimulus inputs of several magnitudes. **b** Transition of firing frequency. The horizontal axis represents the magnitude of stimulus input, and the vertical axis represents the frequency.

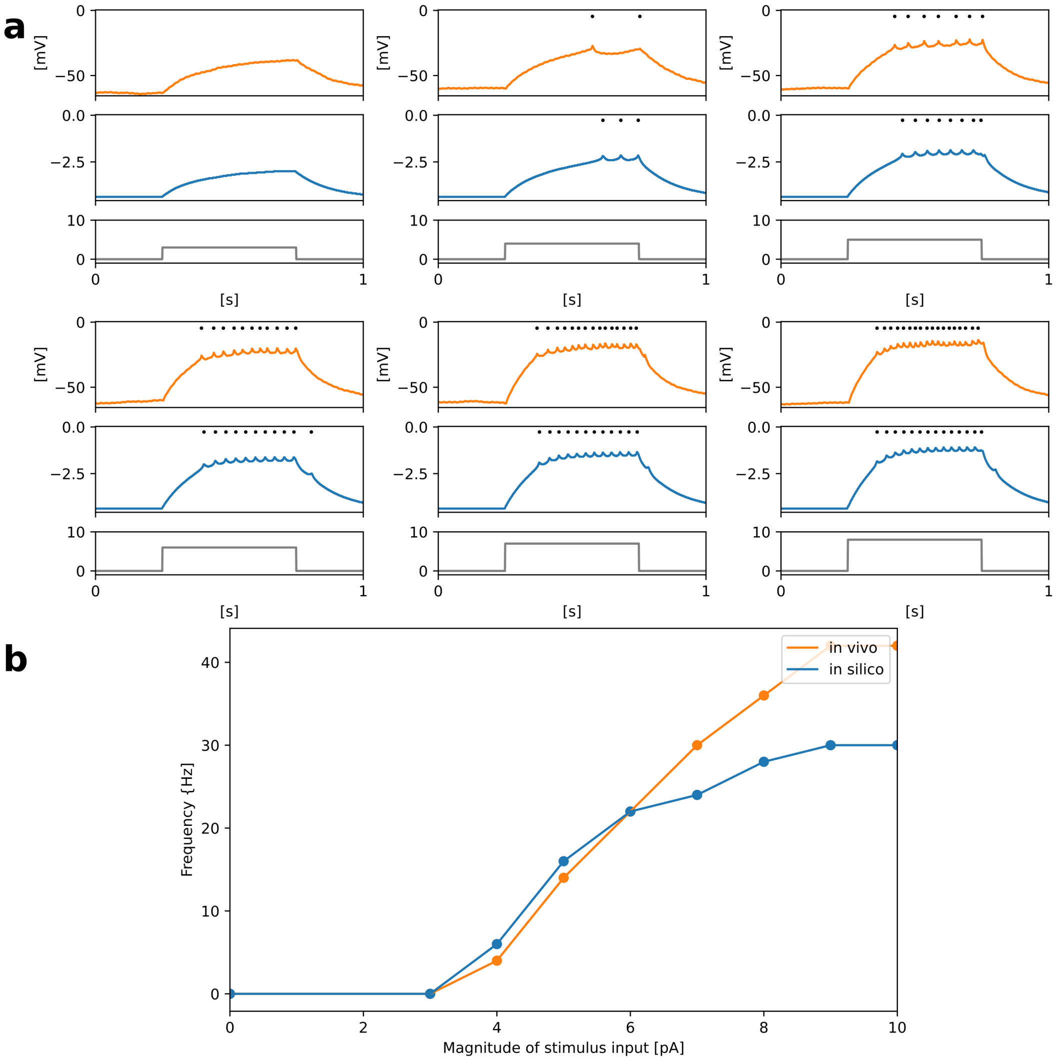

Figure S5. Responses of PN in vivo and in silico. **a** Responses of somatic membrane potentials in vivo (orange) and in silico (blue) in response to step stimulus inputs of several magnitudes. **b** Transition of firing frequency. The horizontal axis represents the magnitude of stimulus input, and the vertical axis represents the frequency.

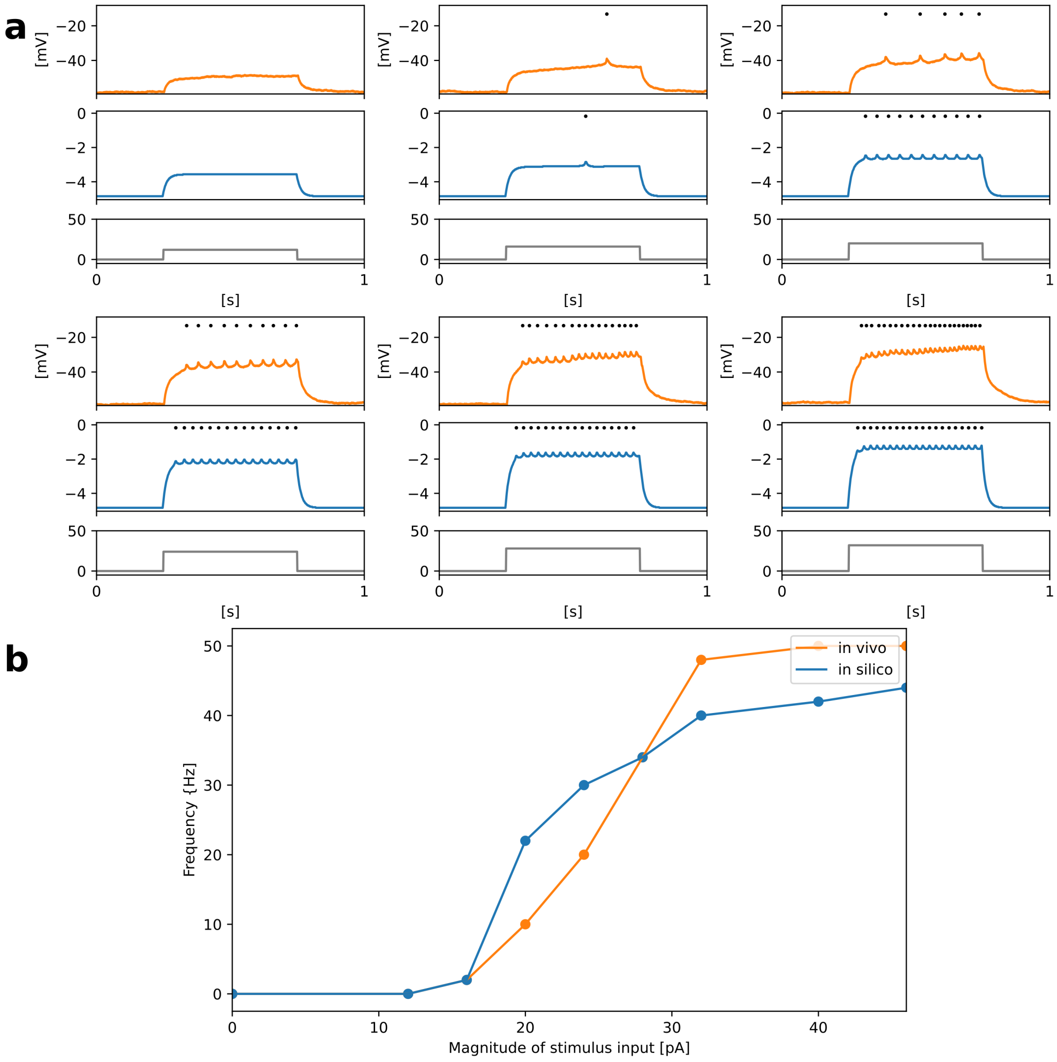

Figure S6. Responses of KC in vivo and in silico. **a** Responses of somatic membrane potentials in vivo (orange) and in silico (blue) in response to step stimulus inputs of several magnitudes. **b** Transition of firing frequency. The horizontal axis represents the magnitude of stimulus input, and the vertical axis represents the frequency.

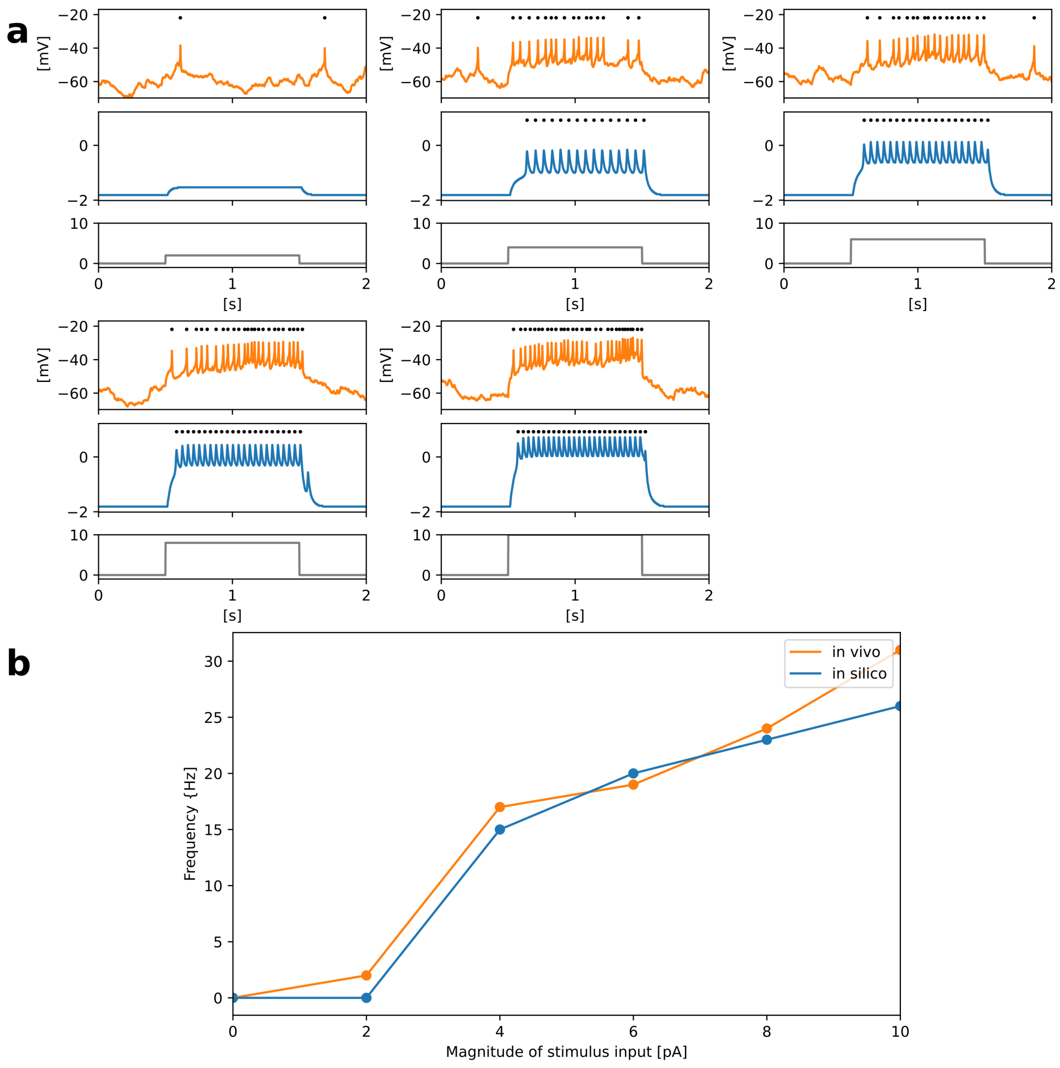

Figure S7. Responses of MBON in vivo and in silico. **a** Responses of somatic membrane potentials in vivo (orange) and in silico (blue) in response to step stimulus inputs of several magnitudes. **b** Transition of firing frequency. The horizontal axis represents the magnitude of stimulus input, and the vertical axis represents the frequency.

2 SUPPLEMENTARY NOTES

2.1 Supplementary Note 1

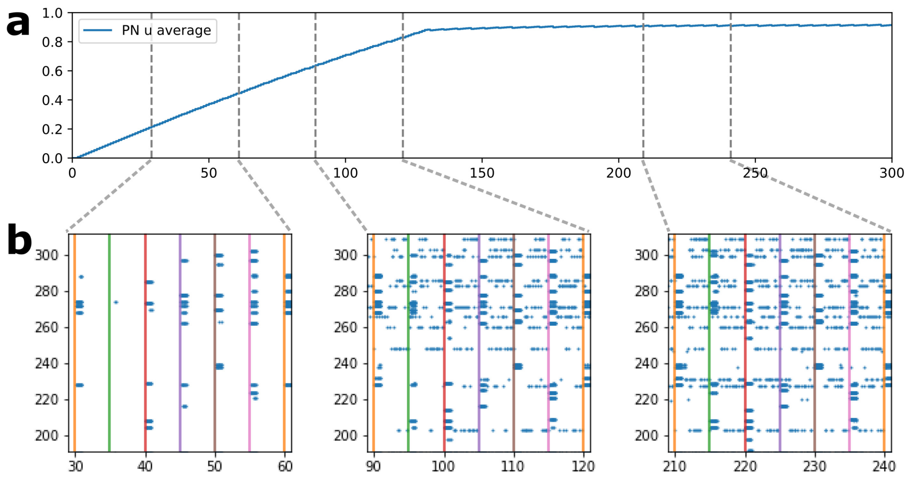

Figure S8. Transition of the averaged value of u for all PNs during the homeostatic period.

Before conducting all the experiments shown in Figures 3–5, 300 seconds of ORN input data were provided to the network to implement the homeostatic control of synaptic input observed in PNs. The data structure was identical to that used for olfactory associative learning, where six odorants, 3-octanol, cis-3-hexenol, cyclohexanone, 2,3-butanedione, 2-hexanol, and ethyl butyrate, were applied sequentially for one second every five seconds. Supplementary Figure S8a shows the transition of the average value of u for all the PNs during this period. The initial value of u is fixed at 0, and as the value gradually increases, a larger number of PNs fire to the odor (Supplementary Fig. S8b).

2.2 Supplementary Note 2

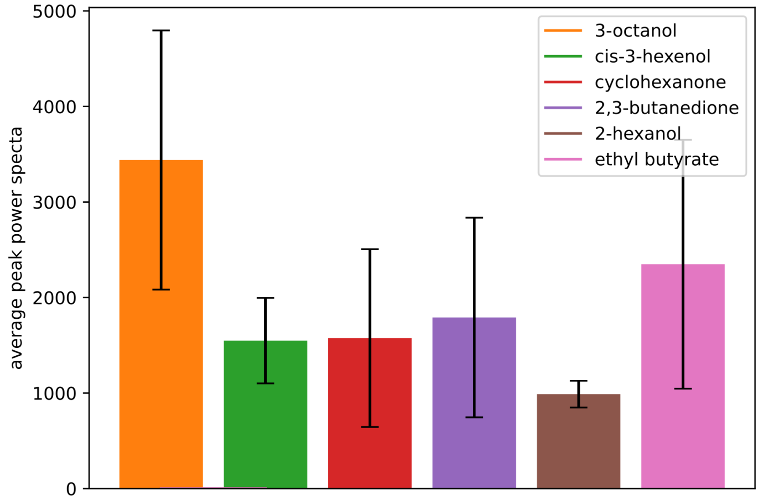

Figure S9. Averages of the peak power spectra of PNs when one of the six odorants, 3-octanol, cis-3-hexenol, cyclohexanone, 2,3-butanedione, 2-hexanol, and ethyl butyrate, was applied. Error bars represent standard deviation over five trials.

2.3 Supplementary Note 3

The fft package of the NumPy module in Python was used to calculate the power spectra of the virtual LFP. Here, only 8 seconds of the 10-second responses of the virtual LFPs to the odor, excluding the first and last seconds, were used to compute the power spectra.

2.4 Supplementary Note 4

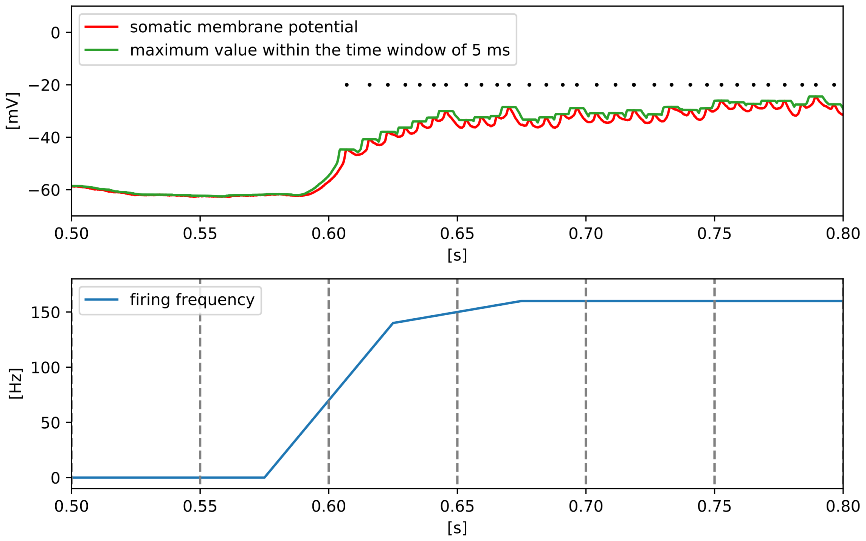

Figure S10. Spike detection and frequency calculation for in vivo data of the somatic membrane potential.

Here, we describe the detection of spike timing from the somatic membrane potentials of in vivo data. The amplitude of spikes observed in the soma is decayed and small. In addition, the baseline of the somatic membrane potential during repeated firings fluctuates significantly. Therefore, we first plotted the maximum value of the membrane potential within the time window of 5 ms at each time point (Supplementary Fig. S10). Here, a spike is detected at a time point when the value of the time point is equal to the maximum value within the time window and the value of the time point is greater than the value of the previous time point. The latter rule prevents the detection of spikes twice when two adjacent maxima of the same value are measured at the top of the spike. Then, for each 50ms, the number of spikes was counted, and the instantaneous firing frequency was calculated as follows:

$$\mathrm{firing\ frequency} = \mathrm{n_0/w_0} \tag{S1}$$

where n_0 is the number of spikes in the 50 ms period and w_0 is 50 ms.

In the simulation, a spike was detected when the value of the membrane potential of the axonal compartment exceeded 0. The calculation of the frequency was performed in the same way as that in the in vivo data.

3 SUPPLEMENTARY TABLES

Table S1. Parameter set for the Krasavietz class1.

Par.	Value	Par.	Value
Δ t	0.001	τ	0.008
afn	1.021484375	afp	-2.3544921875
bfn	-1.09375	cfn	0.93359375
agn	-0.7119140625	agp	9.5595703125
bgn	-4.8232421875	cgn	13.427734375
ahn	-0.7041015625	ahp	8.640625
bhn	-3.6533203125	chn	4.521484375
I_{b0}	5.2509765625	k_I	16.3193359375
ϕ	0.703125	ϵ_q	0.0107421875
r_g	-1.234375	r_h	-1.955078125
m_0	-1	m_1	1

Table S2. Parameter set for the Krasavietz class2.

Par.	Value	Par.	Value
Δ t	0.001	τ	0.008
afn	3.6650390625	afp	-13.3037109375
bfn	-1.4853515625	cfn	4.1650390625
agn	1.6240234375	agp	14.0205078125
bgn	-0.6923828125	cgn	11.5205078125
ahn	-0.603515625	ahp	5.62890625
bhn	-4.0517578125	chn	4.6513671875
I_{b0}	2.236328125	k_I	16.703125
ϕ	0.19921875	ϵ_q	0.01171875
r_g	-1.126953125	r_h	-1.6884765625
m_0	-1	m_1	1

Table S3. Parameter set for the NP1227 class1.

Par.	Value	Par.	Value
Δ t	0.001	τ	0.008
afn	1.126953125	afp	-10.0498046875
bfn	-1.189453125	cfn	-8.6083984375
agn	-1.2724609375	agp	13.484375
bgn	-6.3671875	cgn	2.560546875
ahn	-0.78125	ahp	11.970703125
bhn	-3.76953125	chn	4.0849609375
I_{b0}	-8.587890625	k_I	32.14453125
ϕ	1.3974609375	ϵ_q	0.0107421875
r_g	-1.7109375	r_h	-1.6904296875
m_0	-1	m_1	1

Table S4. Parameter set for the NP2426 class1.

Par.	Value	Par.	Value
Δ t	0.001	τ	0.008
afn	5.279296875	afp	-15.05859375
bfn	-0.94921875	cfn	-1.0498046875
agn	-14.443359375	agp	6.1220703125
bgn	-4.09375	cgn	15.2958984375
ahn	-4.63671875	ahp	-0.97265625
bhn	-3.5986328125	chn	4.21484375
I_{b0}	-13.65234375	k_I	11.9833984375
ϕ	0.5576171875	ϵ_q	0.046875
r_g	-3.203125	r_h	-4.390625
m_0	-1	m_1	1

Table S5. Parameter set for the PN.

Par.	Value	Par.	Value
Δ t	0.001	τ	0.00390625
afn	0.25	afp	-0.25
bfn	-3.0	cfn	0.0
agn	0.125	agp	1.0
bgn	-2.0	cgn	-4.0
ϕ	3	θ	0.0078125
r_g	-0.5	k_I	1
k_0	-0.5	k_1	-4
k_r	12	I_{b0}	-4
I_{b1}	-16	m_0	-1
m_1	1	F_t	1
κ	0.00390625	m_0	-1
m_1	4		

Table S6. Parameter set for the KC.

Par.	Value	Par.	Value
Δ t	0.001	τ	0.00390625
afn	0.25	afp	-0.25
bfn	-3.0	cfn	0.0
agn	-0.25	agp	1.0
bgn	-4.0	cgn	-4.0
ϕ	2	θ	0.0625
r_g	-0.5	k_I	1
k_0	-0.5	k_1	-4
k_r	12	I_{b0}	-4.2
I_{b1}	-19.52	m_0	-1
m_1	1		

Table S7. Parameter set for the MBON.

Par.	Value	Par.	Value
Δ t	0.001	τ	0.00390625
afn	1.0	afp	-0.25
bfn	-1.0	cfn	0.0
agn	-0.25	agp	1.0
bgn	-3.0	cgn	-4.0
ϕ	0.5	θ	0.03125
r_g	-0.5	k_I	4
k_0	-0.5	k_1	-1
k_r	4	I_{b0}	-4.8
I_{b1}	-2.3	m_0	-1
m_1	2		

Table S8. Parameter set for the APL.

Par.	Value	Par.	Value
Δ t	0.001	τ	0.00390625
afn	0.125	afp	-0.125
bfn	-0.0	cfn	0.0
agn	0	agp	1.0
bgn	-0.0	cgn	-4.0
I_{b0}	-5	k_I	1
ϕ	0.125	ϵ_q	0.0078125
r_g	-0.0	r_h	0
m_0	-1	m_1	1

Table S9. Parameter set for the SMP354.

Par.	Value	Par.	Value
Δ t	0.001	τ	0.00390625
afn	0.25	afp	-0.25
bfn	-3.0	cfn	0.0
agn	-0.25	agp	1.0
bgn	-4.0	cgn	-4.0
ahn	0	ahp	0
bhn	0	chn	0
I_{b0}	-4.2	k_I	1
ϕ	4	ϵ_q	0
r_g	-0.5	r_h	0
m_0	-1	m_1	1

Table S10. Values of scaling parameters $p_{x\text{-}y}$.

x	y	Value	x	y	Value
ORN	LN	0.0087890625	ORN	PN	0.125
LN	LN	0.00390625	LN	PN	0.004638671875
PN	LN	1.0	PN	PN	0.125
PN	KC	1.03125	PN	APL	0.078125
KC	MBON-$\alpha 3$	0.3125	KC	MBON-$\alpha 1$	0.5625
KC	APL	0.078125	APL	MBON-$\alpha 3$	1
APL	MBON-$\alpha 1$	1	MBON-$\alpha 3$	SMP354	0.28125
MBON-$\alpha 1$	SMP354	0.3125			
